# Antenatal screening practices in the WHO European Region: a mixed methods study

**DOI:** 10.7189/jogh.10.020416

**Published:** 2020-12

**Authors:** Marzia Lazzerini, Benedetta Armocida, Emanuelle Pessa Valente, Nino Berdzuli

**Affiliations:** 1 Institute for Maternal and Child Health – IRCCS “Burlo Garofolo”, Trieste, Italy; 2 Division of Noncommunicable Diseases and Promoting Health through the Life-course, WHO Regional Office for Europe, UN City, Copenhagen, Denmark

## Abstract

**Background:**

Literature suggests an increasing trend towards more screening tests, while awareness of potential harms of screenings has been reported to be sub-optimal. This paper aimed to characterize ANC screening practices within the 53 countries of the WHO Europe Region and compare these to evidenced-based recommendations from WHO and from other key reference sources.

**Methods:**

From January 2019 to July 2019 we conducted a survey among key informants (KIs) in the 53 countries of the WHO European Region and a systematic review of literature. KIs were invited to answer an online structured questionnaire, available both in English and Russian. Published and unpublished guidelines, policies or cross-sectional studies on ANC screening practices were searched for in four electronic databases (MEDLINE, Global Health Library, Web of Science, Google) and also sent by KIs. Data obtained from both methods were analysed and triangulated by two independent authors.

**Results:**

Overall 42 countries participated in the survey. Among these, 36 (86%) reported national guidelines on ANC screening, but only 26 (61.9%) reported up-to-date and comprehensive guidelines. All countries reported supplemental use other guidelines, with 19 (45.2%) using more than three. When looking at current evidence-based recommendations, only one (ultrasound before 24 weeks) was reported to be implemented in all countries. Overall, 35 (83.3%) countries reported using at least five not-recommended ANC screening practices, with 21 (50%) implementing ≥10 not-recommended ANC screening practices. The systematic review resulted in 11871 records, with 111 (90 guidelines, 4 policies, 17 cross-sectional studies) matching inclusion criteria. Findings from the systematic review were largely consistent with those of the online survey: among the most comprehensive national guidelines identified, only six (24%) had a concordance ≥75% with the reference recommendations, independently from their publication date, while the few existing cross-sectional studies highlighted large heterogeneity in the implementation of ANC practices among countries.

**Conclusions:**

Guidance on and implementation of evidenced-based recommendations on ANC screening is suboptimal in the WHO European Region. It is necessary to increase the availability of evidence-based high-quality national guidelines and their concrete use in routine practice.

The World Health Organization (WHO) establishes quality of care as fundamental for Maternal and Child Health [1,2], and essential for the health and well-being of the population, and as a basic aspect of human rights [3,4].

Medical screening detects previously undetected diseases/conditions in individuals and populations. Although there is not a single and complete definition [5-7], screening is usually defined as the likely identification of unrecognized disease/condition in an apparently healthy, individual or population by means of tests, examinations or other procedures that can be applied rapidly and easily to the target population [7].

Benefit/harm profiles for screening tests vary widely and depend on a range of factors. Assessment of benefit/risk is sub-optimal in the implementation and interpretation of screening tests, a growing problem as testing rates increase.

Risks posed by screening tests is misdiagnosis. First, false-positive results can lead to unnecessary psychological distress, investigations and treatments, while false-negative results can create a false sense of security and may delay diagnosis [8-10]. Second, screening tests can also lead to overdiagnosis, where a positive result is correct but irrelevant because effective treatment is not available or symptoms are unlikely to arise during the patient’s lifetime [8,9]. Third, beside potential individual harm, screening can increase costs both for the health system and the community [9]. While there is a growing global trend towards more health screening, awareness of the potential harms of these tests among policy makers, health professionals and the public has been reported to be sub-optimal [11-17]. Consequently, screening tests can be both inappropriately under-used or overused [8,9].

Although, the latest WHO guideline on antenatal care (ANC) for a positive pregnancy experience [18] includes ANC screening practices, there is a gap on the actual use and implementation of these in the European Region. We hypothesized that there might be heterogeneity in ANC screening practices within the Region that has not been recently reported in the literature. Hence, this mixed methods study aimed to characterize ANC screening practices within the 53 countries of the WHO Europe Region and compare these to evidenced-based recommendations from WHO and from other key reference sources. The results of this study could be used to better direct the implementation of recommended guidelines on ANC within the Region, to strengthen its monitoring mechanisms, and to develop recommendations more feasible to various settings.

## METHODS

### Study design

We used a mixed methods design. First, we selected evidenced-based reference recommendations on ANC screening from WHO and other key sources. Second, we developed a pilot-tested structured online survey, which was administered to key informants (KIs). In parallel, we performed a systematic review of existing literature, including grey literature (unpublished reports), to retrieve any record describing ANC screening practices in the 53 countries within the WHO European Region. Data resulting from both sources were used to characterize ANC screening practices in the WHO European Region and compare them to the identified reference recommendations.

Both the Standards for Reporting for Qualitative Research (SRQR) [19] and the PRISMA (Preferred Reporting Items for systematic reviews and meta-analyses) statement [20] (Appendices S1 and S2 in the **Online Supplementary Document**) were applied to reporting of study results.

### Selection of reference recommendations

In line with existing definitions of medical screening [5-7], we defined ANC screening as “systematic application of a laboratory tests and/or equipment or medical devices for identification of an unrecognized disease/condition in an apparently healthy, pregnant woman”.

The primary source of reference recommendations was selected based on its up-to-date (published in the last 5 years), producer and methodology (developed by an intergovernmental agency following well defined criteria for guideline development), and comprehensiveness (including overarching ANC screening practices recommended in European countries), by two authors (ML, EPV), experienced in evidence synthesis in maternal and newborn care. Any discordance was solved by consensus with a third author (NB). If the topic was not covered by of the primary source of reference, a secondary source of reference was sought. Finally, the ANC screening practices were defined as “recommended” or “not recommended”.

### Online survey

We conducted an online survey using a structured pilot-tested questionnaire among KIs to identify ANC screening practices recommended and implemented at country level. KIs with senior expertise in maternal health were identified by a) searching the websites of key national organizations and bodies such as the Ministry of Health (MoH), National Centers of Research in Maternal Health or Evidence-Based Medicine (EBM), and Scientific Societies; b) literature review to identify authors of the most relevant studies; and c) personal contacts with experts. All were invited to participate and to those providing consent and confirming a good comprehension of the English and/or Russian language were administered the survey. Participants were also invited to distribute the survey link to other national experts with expertise in the topic. A personalised link to the survey were sent via email, reminder emails were sent every two weeks after the initial invitation. Google forms survey tool was used (Google Inc.).

The structured questionnaire consisted of 15 multiple choice questions and 5 open questions, with the option of sending relevant national documents that might confirm their responses, with no language and date limitation. The estimated completion time was of 10-12 minutes (Appendix S3 in the **Online Supplementary Document**).

Initially, it was qualitatively evaluated through face and content validity by a panel of experts with extensive experience in maternal health. Specifically, face validity was performed through expert’s assessment of items to evaluate the appearance of the questionnaire in terms of feasibility, readability, consistency of style and formatting, and the clarity of the language. Content validity was assessed in order to ensure that all essential items were included and undesirable items eliminated. Content validity was conducted through a comprehensive literature review to extract the items, each item was cross checked with the reference recommendations, and the questionnaire was subsequently sent to a panel of experts in the field. A preliminary version was piloted in a sample of ten experienced obstetricians and gynaecologists. The questionnaire was developed in English and professionally translated in Russian. The translation consisted in forward and backward translation, and semantic, idiomatic and conceptual discrepancies were resolved. Both questionnaires were administered during the period 15th January to 30th April 2019. Overall, 229 experts from 53 countries were contacted. Professional characteristics of KIs are reported in Appendix S4 in the **Online Supplementary Document**. In case of missing data, unclear responses, and potential major discrepancies among KIs from the same country/geographical area, further clarification was sought from KIs.

### Literature review

We searched MEDLINE through PubMed (from 1956), the Global Health Library (WHO website), and the Science Citation Index Expanded (SCI-EXPANDED) and the Social Sciences Citation Index (SSCI) through Web of Science for guidelines, policies or cross-sectional studies related to ANC practices in any of the 53 WHO European Region countries (detailed search strategy in Appendix S5 in the **Online Supplementary Document**). The initial search was conducted on 7th January 2019, updated on 30th July 2019, and limited to records published on or after 1st January 2015. Additionally, we searched for grey literature using Google, assessed documents shared by KIs during the online survey, hand-searching of the title on reference lists was also performed.

#### Eligibility of articles for inclusion in the review was assessed using criteria established a priori.

Eligible articles reported on a) national or subnational guideline/consensus statements/policies on any type of ANC screening from one or more of the 53 countries of the WHO European Region and/or b) national cross-sectional studies on implementation of ANC screening practices (surveys or observational studies). We considered both stand-alone ANC screening guidelines and those that were part of overarching maternal or prenatal care guidelines. Moreover, in case that more than one guideline was found for a specific country we only included the most recent publication. Observational studies reporting on practices from a single facility were not included, as they could not be considered representative of the country. Experimental studies exploring new screening methods/strategies or opinion papers were not included, since they cannot represent the average practice in the country/Region. Abstracts for which full text was not available were not included. Furthermore, in the literature review from the four electronic database we excluded all the non-English language, while the relevant national documents shared by KIs had no language limitations. Those of which the language was other than English or Italian or Spanish or Dutch or Portuguese or Russian or Georgian or Lithuanian or Danish or Swedish (from which native experts had facilitated the translation) were translated via Google Translate. The content of translation was compared with the online survey responses of KIs from the same country/geographical area, and verified against the original versions by expert native speakers.

Two independent authors (ML, BA) reviewed articles for eligibility, reviewing the full text of articles deemed potentially eligible based on review of the title or abstract. Disagreement between reviewers was resolved by consensus.

The full text of all eligible citations, including grey literature and national relevant documents sent by KIs, was examined independently and in detail by two authors (BA, EPV) who extracted data using a pre-piloted data-extraction form, through which the quality of each paper was assessed. Any disagreements were resolved by consensus between the two authors and if necessary with a third author (ML). For each cross-sectional study, we expressed the implementation level reported on the publication as percentage of national/regional coverage of the ANC screening practices.

The recommendations provided in each guideline were compared with the primary and secondary sources of reference recommendations. Screening reported in guidelines as “on the horizon” were also documented. As heterogeneity of the resulting data did not allow metanalysis, we reported data in tables and text.

### Ethical aspects

The study did not imply any experiment or intervention in human subjects. The systematic review did not require any formal ethical clearance. For the online survey, study objectives and methods were explained to KIs, and written informed consent was sought prior to participation. Data collected were completely anonymized. No detail is reported that could reveal the identity of participants in the survey.

## RESULTS

### Selected reference recommendations

Based on these criteria, we defined the WHO recommendations for a positive pregnancy experience [18] as the primary source of reference recommendations. As secondary sources we referred to the National Institute for Health and Care Excellence (NICE) (ANC for uncomplicated pregnancies, diabetes, hypertension and routine antenatal anti-D prophylaxis in pregnancy) [21-24] or the Royal College of Obstetricians and Gynecologists (RCOG) (thromboembolic disease in pregnancy) [25] or other accredited European association [26]. A comparison among the selected reference recommendations is reported in Appendix S6 in the **Online Supplementary Document**.

Based on the source of reference recommendations, we identified 16 “recommended” and 16 “not recommended” practices ANC screening practices. A detailed description of these practices and the source of each are reported in Appendices S7 and S8 in the **Online Supplementary Document**.

### Results of the online survey

#### Survey responses and geographic coverage

Overall, KIs from 42 countries participated in the survey. These included countries from different sub-regions, including: Northern, Western, Eastern and Southern Europe, and Central Asia.

#### Characteristics of ANC guidelines

Out of the 42 countries with available information, the existence of official national guidelines was reported for 36 (85.7%). However, national guidelines were comprehensive and updated based on recent evidence in only in 26 (61.9%) countries (Appendix S9 in the **Online Supplementary Document**).

**Figure 1:**
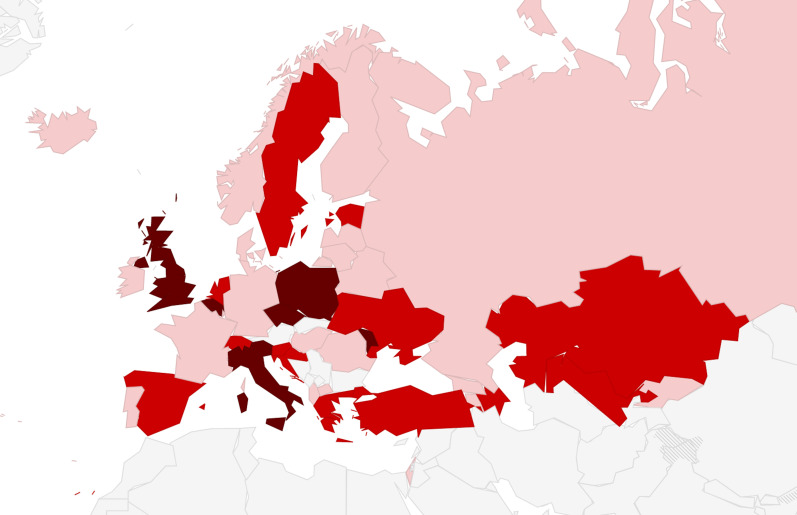
Number of different guidelines on ANC screenings reported as used in clinical practice. Legend: pink − from 2 to 3 guidelines; light red − from 3 to 5 guidelines; dark red − more than 5 guidelines. Note: the question specified different guidelines from different sources, such as guidelines from different organizations such as different scientific societies.

In 30 (71.4%) countries, national guidelines were reported to be widely used, while in seven (16.7%) they were not, and in the remaining five (11.9%) guideline diffusion was unclear. All countries were reported to be using guidelines from different sources in addition to official national guidelines, with 19 (45.2%) using more than three different guidelines (**Figure 1**). Specifically, the following other guidelines were used: guidelines of scientific societies/organization such as NICE, RCOG (27%); guidelines from local scientific societies (26%); WHO guidelines (17%). Overall 14 (33.3%) countries reported that the degree of heterogeneity among recommendations in different guidelines adopted was “medium”, while a KI in one country rated it as “high”. Detailed data are provided in Appendix S9 in the **Online Supplementary Document****.**

#### Implementation of reference recommendations

**Figure 2:**
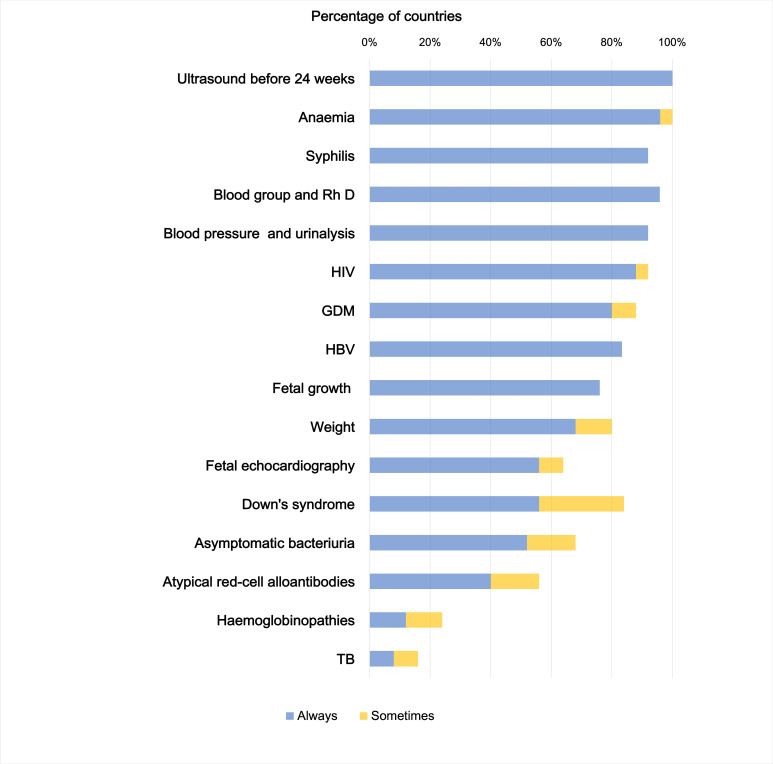
Reported implementation of “recommended” ANC screenings. Abbreviations: GDM − gestational diabetes mellitus; HIV − human immunodeficiency virus; HBV − hepatitis B virus; Rh D − rhesus D; TB − tuberculosis.

Only one, ultrasound (US) before 24th week, of the 16 “recommended” ANC screening practices (Appendix S7 in the **Online Supplementary Document**) was reported as implemented in 100% of countries (**Figure 2**). No country reportedly “always” implements all recommendations.

**Figure 3:**
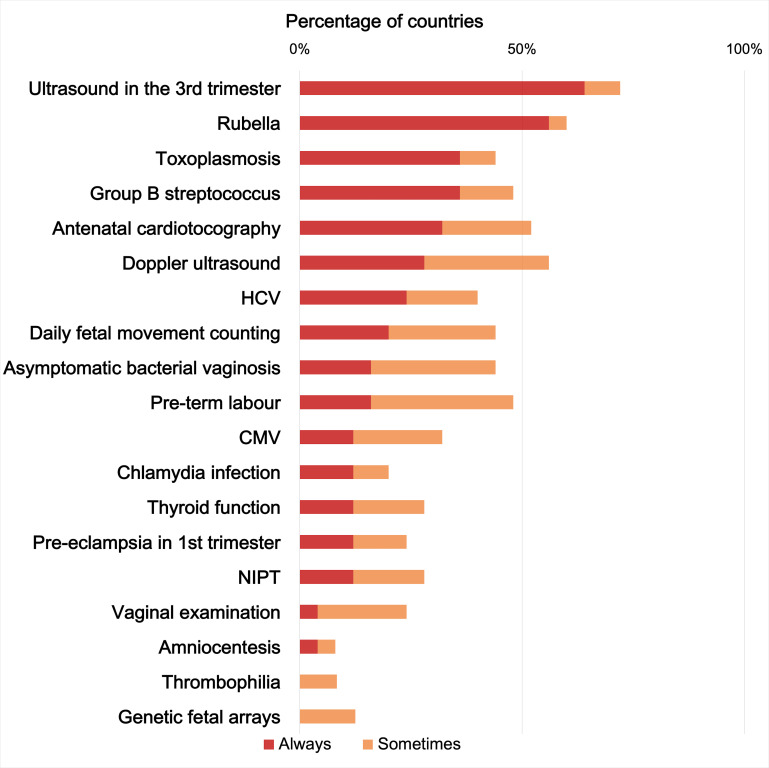
Reported implementation of “not-recommended” ANC screenings. Abbreviations: CMV − cytomegalovirus; HCV − hepatitis C virus; NIPT − noninvasive prenatal testing.

In contrast, several of the 16 “not recommended” ANC screening practices (Appendix S8 in the **Online Supplementary Document**) were widely implemented in most countries. Specifically, 42 (100%) countries implemented at least one of the “not recommended” ANC screenings; 35 (83.3%) used at least five of the “not recommended” practices in routine care, and among these 21(50%) used 10 or more “not recommended” practices, with the most frequent being 3rd trimester US and screenings for rubella and toxoplasmosis (**Figure 3**).

Additionally, nine other screening practices were utilized (Appendix S10 in the **Online Supplementary Document**), while 11 other tests were reported as being considered (ie, “on the horizon”) (Appendix S11 in the **Online Supplementary Document**).

**Figure 4:**
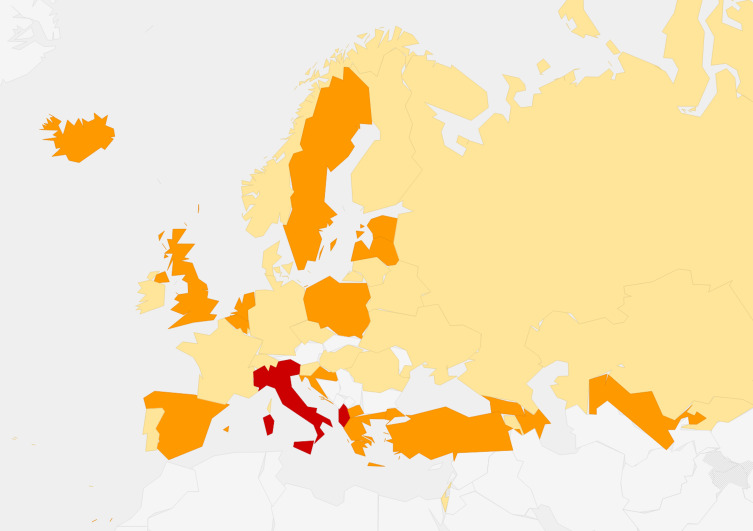
Degree of within-country heterogeneity in the ANC screenings. Legend: yellow − low; orange − medium; red − high.

A medium to high level of heterogeneity among ANC screening practices in hospitals/institutions within the same country was reported by 19 (45.2%) countries (**Figure 4**). Overall 15 explanations were posited for this heterogeneity, the top four being: “different practice in public health services vs. private” (33.3%); “fear of litigation” (26.2%); “practice based on tradition” (23.8%); and “lack of supervision” (21.4%) (Appendix S12 in the **Online Supplementary Document**).

ANC screening practices changed in the recent past in over a third of countries, with 16 (38.1%) reporting that at least one ANC screening practice had been suspended in the last 15 years (Appendix S9-Panel F in **the** **Online Supplementary Document**). Detailed data are provided in Appendix S13 in the **Online Supplementary Document****.**

### Results of the systematic review

**Figure 5:**
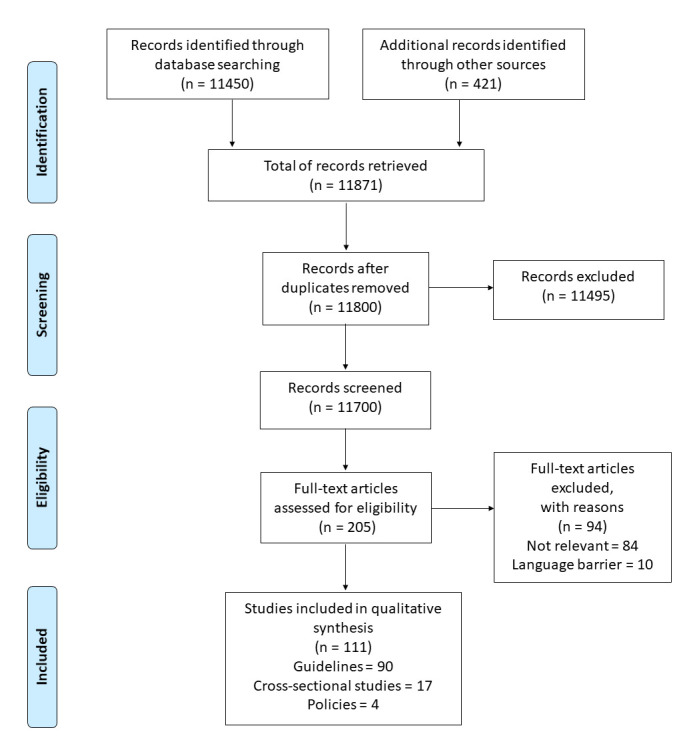
Systematic review flow diagram.

The systematic review resulted in 11871 records, among which 111 (90 guidelines, 4 policies, 17 single studies) matched the inclusion criteria (**Figure 5**). The full reference list is provided in Appendix S14 in the **Online Supplementary Document**.

Number of records identified varied significantly by geography, with countries from Northern and Western Europe contributing higher numbers of references (16 records retrieved from United Kingdom [UK], 10 from Switzerland, seven from Ireland, six from Denmark, Germany and Norway). Further detailed are provided in Appendix S15 in the **Online Supplementary Document**. A total of 34 national references were identified through KIs.

#### Characteristics of ANC guidelines

A total of 90 guidelines were retrieved and classified in three categories according the number and type of topic considered: multiple topics (n = 25), single topics (n = 52), and guidelines on topics not included in our reference sources (n = 13). Only 62 (68.8%) guidelines were recently updated (ie, during or after 2015). While nearly all (92%) multiple topic guidelines were produced by an MoH, topic specific guidelines were mostly published by scientific societies (46.2%) (Appendix S16 in the **Online Supplementary Document**).

**Figure 6:**
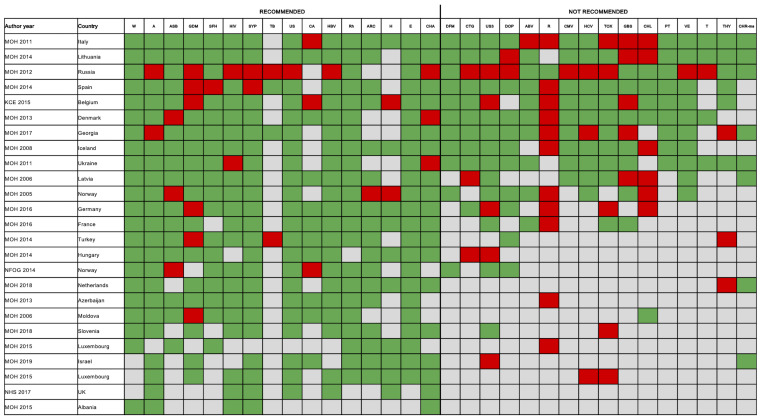
Concordance between national ANC guidelines identified by the systematic review and reference recommendations. Legend: green − in-line; red − not in-line; grey − not covered. Abbreviations: UK − United Kingdom, W − weight, A − anaemia, ASB − asymptomatic bacteriuria, GDM − gestational diabetes mellitus, SFH − fetal growth, HIV − human immunodeficiency virus, SYP − syphilis, TB − tuberculosis, US − gestation age, multiple pregnancies and fetal anomalies ultrasound, CA – cardiac anomalies, HBV – hepatitis B virus, Rh − alloimmunization, ARC − atypical red-cell alloantibodies, H − haemoglobinopathies, E – pre-eclampsia, CHA − Chromosomal abnormalities, DFM − daily fetal movement counting, CTG – antenatal cardiotocography, US3 − fetal growth, DOP − Doppler ultrasound of fetal blood vessels, ABV − asymptomatic bacterial vaginosis, R – rubella, CMV − cytomegalovirus, HCV – hepatitis C virus, TOX − toxoplasmosis, GBS − group B streptococcus, CHL − *Chlamydia trachomatis*, PT - pre-term labour, VE − vaginal examination, T − thrombophilia, THY − thyroid function, CHA-ms − chromosomal abnormalities by morphological scan.

**Figure 6** depicts the concordance between 25 multiple topic national guidelines and the reference recommendations. The number of topics covered by each guideline ranged from 5 to 30, with those covering the greatest number of ANC screenings produced by Italy, Lithuania and Russia (Appendix S17 in the **Online Supplementary Document**) and only 13 guidelines covered at least 50% of the topics included in reference recommendations. None of the guidelines were fully in line with the reference recommendations. Overall, only six (24%) guidelines (Denmark, Iceland, Italy, Lithuania, Spain, Ukraine) included were concordant with at least 75% of the 32 reference recommendations, with those produced by Lithuania being the most comprehensive and in line (81% of concordance) (**Figure 6**). A recent year of publication did not correlate with the percentage of topics in line with the reference guidelines (*P* = 0.125, R^2^ = 0.09) (Appendix S18 in the **Online Supplementary Document**). The 25 guidelines included a total of 483 ANC screening recommendations (310 “recommended” and 175 “not recommended”). Of the 310 recommended screenings, 281 (90.6%) were in line with the reference guidelines, while among those not recommended 128 (73.1%) were in line with reference guidelines. The most frequently unaligned recommendations were related to gestational diabetes, rubella and *Chlamydia trachomatis*.

Among the 52 topic-specific guidelines, the majority (86.5%) of recommendations were in line with the reference guidelines. The most concordant ANC screening practices were those for anaemia, alloimmunization, cardiotocography, thrombophilia, pre-eclampsia, chromosomal abnormalities, while the least were those for thyroid, infectious diseases, gestational diabetes and US (Appendix S19 in the **Online Supplementary Document**).

The systematic review also identified ANC screenings “on the horizon”. These pertained to three groups: non-invasive prenatal testing, alternative ultrasound techniques or laboratory markers for detecting pre-eclampsia, and tests for infectious diseases not included in the reference practices (Appendix S20 in the **Online Supplementary Document**).

Of the four policies shared by KIs, two were part of an overarching maternal and prenatal care policy for Europe, one on maternity care in Scotland and one on gestational diabetes in Portugal. All policies were published before 2011 (Appendix S14 in the **Online Supplementary Document**), and for this reason they were not further analyzed.

#### Implementation of reference recommendations

Among the 17 cross-sectional studies identified by the systematic review, none was comprehensive and recent: most studies were single-country (82.3%), and most (75%) covered a single ANC screening practice, namely: chromosomal abnormalities (5), diabetes (4), ultrasound (2), and syphilis (1) (Appendix S21 in the **Online Supplementary Document**). About two thirds (64.7%) were produced by research groups without direct endorsement of any society/organization/institution (Appendix S21 in the **Online Supplementary Document**). The majority (58.9%) used indirect methods (eg, questionnaire to health workers) to measure practices. Overall, studies highlighted a large heterogeneity in the implementation of ANC screening practices among countries, with a reported coverage of “recommended” practices ranging from 33% to 100%, and coverage of “not recommended” practices ranging from 24.1% to 98% (Appendix S22 in the **Online Supplementary Document**). As a key example of “recommended” practices, two studies on gestational diabetes conducted in Belgium and the UK highlighted that 67% and 81% of facilities, respectively, performed screening in accordance with reference recommendations (Appendix S22 in the **Online Supplementary Document**). As a key example of “not recommended” practices, two studies on infectious disease screening conducted in Switzerland and in several European Union (EU) countries highlighted that the majority (98%) of clinicians in Switzerland implemented routine group B streptococcus screening and that over half (53.8%) of EU countries implemented rubella susceptibility screenings, both “not recommended” practices.

### Triangulation and interpretation of findings

Overall, findings of the online survey were confirmed by the systematic review. Both the online survey and the systematic review pointed out that despite the existence of official national guidelines in most countries, only 61.9% (according to the online survey results) and 68.8% (according to the systematic review) were recently updated (ie, before 2015) (Appendices S9 and S16 in the **Online Supplementary Document**).

When analyzing results by country, findings of the systematic review, regarding the comprehensiveness of content of guidelines, matched the perspective of KIs. For example, according to the systematic review, the guidelines from Lithuania, Denmark and Spain were in line (81%, 75% and 75%) with the primary and secondary sources of reference recommendations, and these data were assured by the respective national KIs (**Figure 6**; Appendix S17 in the **Online Supplementary Document**).

When considering ANC screening practice implementation, again the online survey was confirmed by data from the systematic review, although existing studies were limited. For example, KIs reported a coverage of diabetes screening around 85%, while studies identified in the systematic review showed a coverage ranging from 64.3% in a multi-country evaluation to 82% in Italy (**Figure 2**; Appendix S22 in the **Online Supplementary Document**).

## DISCUSSION

Findings of this study strongly suggest that, in the WHO European Region, there are gaps in the availability of comprehensive and updated national guidelines on ANC screening. More importantly, implementation of evidenced-based ANC screening recommendations was found to be suboptimal, with significant heterogeneity in practices both between and within countries. We could not retrieve any recent (2015 or more recent) survey on ANC screening practices in the 53 countries of the WHO Region, despite conducting a wide search of electronic databases, contacting a list of experts, and working to identify grey literature. The only comprehensive previous survey on ANC screening practices in this region was conducted in 2004 and limited to only 25 countries within the EU [27]. Findings of the previous survey [27] showed similar results to our survey and review, with relatively good availability of national guidelines, but large heterogeneity among countries both in types of ANC screening recommendations and in practices [27]. A survey conducted by the WHO in 1981 [28] similarly showed that 78% of the 23 participating European Countries had official ANC screening guidelines, suggesting that the production of national guideline is not enough to ensure harmonization of practices, and other actions should be considered and implemented to improve ANC screening in the WHO European Region.

Another interesting finding of this study are the differences found in guidelines on ANC screening among countries [18]. This may be due to use of different criteria when deciding whether to recommend a specific intervention. For example, WHO guidelines^17^ adopt the DECIDE framework [29], a tool that includes explicit and systematic consideration of evidence on interventions in terms of six domains: effects, values, resources, equity, acceptability and feasibility. In the absence of a formal framework, the manual on developing NICE guidelines recommends appraisal of effectiveness, safety and cost of interventions, as well as social values, rather than simply efficacy [30]. In many other guidelines produced by different bodies, criteria supporting final recommendations are not explicit, suggesting a potential role of subjective judgment when translating evidence into recommendations. Additionally, evidence in relation to most of these criteria (ie, harms, values, cost and resources, equity, acceptability and feasibility) is scarce. For example, few studies documented the potential harm and cost related to over-use of ANC screening practices [12], while most national guidelines generally do not emphasize these aspects [31].

Geopolitical differences in ANC screening guidelines may also be justified by heterogeneity in the settings were guidelines are supposed to be used. However, previous studies showed that guidelines can present considerable differences even among countries with similar conditions/settings: for example, one study highlighted that the number of ANC screening practices recommended in each country did not directly link to its economical wealth (measured by the gross national product per capita adapted to the respective national price level according to the purchasing power parity) [27].

Many grey areas still exist in the literature on a number of ANC screening practices. For example, the WHO guideline [18] identified a list of 34 priority questions related to ANC screening (Appendix S23 in the **Online Supplementary Document**), and many WHO recommendations are accompanied by the caveat that more information is needed, with the recommendation either likely to be changed soon, or provisional [18].

We acknowledge that publication bias may have reduced the number of guidelines that we were able to retrieve, although we used a broad search strategy, including grey literature through Google. Additionally, we explicitly requested to KIs to send us the national relevant documents, and they contributed with 34 documents. In general, more research on possible harms, values, cost, equity, acceptability and feasibility of ANC screenings is needed. Criteria for developing recommendations on screenings need to be made more comprehensive and explicit by all bodies producing guidelines. WHO recommendations should be appropriately adapted and reflected in National guidelines. On the other hand, aspects determining heterogeneity in recommendation, such as difference across countries in resources, acceptability and feasibility and values, shall be better acknowledged and addressed in global recommendations.

As highlighted in a *Lancet* series on medical underuse and overuse [32] “full consideration of potential levers of change must include an upstream perspective - ie, an understanding of the system-level factors that drive overuse and underuse, as well as the various incentives at work during a clinical encounter”. Moreover, efforts to increase public awareness on screenings, and community empowerment should be considered as universal methods to promote appropriate care.

We acknowledge limitations for online surveys. Although we carefully selected KIs based on their role, good comprehension of English and/or Russian language and expertise in the maternal field, the respondent KIs might represent a convenience sample. There was also a degree of response bias, and misrepresentation of some part of the WHO Regional Office for Europe, such as Balkan countries, from which we had a low response rate. Moreover, being a self-administered survey there may have been unreported errors among KIs, and although a good knowledge of the selected languages of the survey was required, KIs may have not comprehensively understood some questions. However, other published surveys, including a WHO survey on policies for adolescent health and global survey, used similar methods [33,34]. As in those studies, we sought to validate findings of the survey by also performing a large literature review, and overall findings of the systematic review largely confirmed those of the survey. Other methods for assessing ANC screening practices, such as a direct evaluation, may have the potential advantage of producing more reliable results, but are much more resource intensive and take much longer to make findings available, delaying possible actions in favor of improving ANC screening practices across the Region.

This study highlights a large heterogeneity of ANC screening practice implementation both between different countries and within the same country. The results suggest that monitoring mechanisms should be strengthened, and considerations should be made regarding feasibility of recommended practices across various settings.

## Additional material

Online Supplementary Document
