# Supplementary material for: Antenatal screening practices in the WHO European Region: a mixed methods study
Source: J Glob Health. 2020 Dec 6;10(2):020416. doi: 10.7189/jogh.10.020416 (PMC7719277; doi:10.7189/jogh.10.020416)
Supplement: Online Supplementary Document [file jogh-10-020416-s001.pdf]

Antenatal screening practices within the WHO European Region: a mixed methods study

SUPPLEMENTARY MATERIAL

Table of Contents

S1 Appendix. Standards for Reporting Qualitative Research (SRQR) 3

S2 Appendix. PRISMA checklist 6

S3 Appendix. Questionnaire used for the online survey 9

S4 Appendix. Professional characteristics of KIs 17

S5 Appendix. Search strategies for the literature review 18

S6 Appendix. ANC practices compared between WHO and other reference guidelines 21

S7 Appendix. Details on the "recommended" ANC screenings by reference sources 23

S8 Appendix. Details on the "not recommended" ANC screenings, by reference sources 29

S9 Appendix. Findings of the online survey related to existence, type, and use of guidelines..... 32

S10 Appendix. Other examples of ANC screening utilised at country level, reported by KIs 34

S11 Appendix. ANC screening practices "on the horizon", as reported by KIs 35

S12 Appendix. Factors affecting heterogeneity of ANC screenings, as reported by KIs 36

S13 Appendix. ANC screenings suspended in the last 15 years, as reported by KIs 37

S14 Appendix. References included in the systematic review 38

S15 Appendix. Records included in the systematic review, by country 45

.. 45

S16 Appendix. Characteristics of guidelines on ANC screenings identified by the systematic review (N=90) 46

S17 Appendix. Characteristics of multi-topic guidelines identified by the systematic review and comparison with reference recommendations 47

S18 Appendix. Correlation among concordance rate and year of publication of multi-topic guidelines identified by the systematic review 49

S19 Appendix. Characteristics of topic-specific guidelines identified by the systematic review and comparison with reference recommendations 50

S20 Appendix. ANC practices identified as "on the horizon" by the systematic review 53

S21 Appendix. Number of cross-sectional studies on ANC screening practices identified by the systematic review (N=17) 54

S22 Appendix. Characteristics of cross-sectional studies on ANC screening practices identified by the systematic review 55

S23 Appendix. WHO Research Priorities on ANC 60

S1 Appendix. Standards for Reporting Qualitative Research (SRQR)

Source: http://www.equator-network.org/reporting-guidelines/srqr/

Page/line no(s).

Title and abstract

	**Title** - Concise description of the nature and topic of the study Identifying the study as qualitative or indicating the approach (e.g., ethnography, grounded theory) or data collection methods (e.g., interview, focus group) is recommended	1
	**Abstract** - Summary of key elements of the study using the abstract format of the intended publication; typically includes background, purpose, methods, results, and conclusions	2-3

Introduction

	**Problem formulation** - Description and significance of the problem/phenomenon studied; review of relevant theory and empirical work; problem statement	5
	**Purpose or research question** - Purpose of the study and specific objectives or questions	6

Methods

	**Qualitative approach and research paradigm** - Qualitative approach (e.g., ethnography, grounded theory, case study, phenomenology, narrative research) and guiding theory if appropriate; identifying the research paradigm (e.g., postpositivist, constructivist/ interpretivist) is also recommended; rationale**	9-10
	**Researcher characteristics and reflexivity** - Researchers' characteristics that may influence the research, including personal attributes, qualifications/experience, relationship with participants, assumptions, and/or presuppositions; potential or actual interaction between researchers' characteristics and the research questions, approach, methods, results, and/or transferability	NA
	**Context** - Setting/site and salient contextual factors; rationale**	9
	**Sampling strategy** - How and why research participants, documents, or events were selected; criteria for deciding when no further sampling was necessary (e.g., sampling saturation); rationale**	9-10
	**Ethical issues pertaining to human subjects** - Documentation of approval by an appropriate ethics review board and participant consent, or explanation for lack thereof; other confidentiality and data security issues	14
	**Data collection methods** - Types of data collected; details of data collection procedures including (as appropriate) start and stop dates of data collection and	9-11

	analysis, iterative process, triangulation of sources/methods, and modification of procedures in response to evolving study findings; rationale**	
	**Data collection instruments and technologies** - Description of instruments (e.g., interview guides, questionnaires) and devices (e.g., audio recorders) used for data collection; if/how the instrument(s) changed over the course of the study	9,10; S3 Appendix
	**Units of study** - Number and relevant characteristics of participants, documents, or events included in the study; level of participation (could be reported in results)	9-10; S4 Appendix
	**Data processing** - Methods for processing data prior to and during analysis, including transcription, data entry, data management and security, verification of data integrity, data coding, and anonymization/de-identification of excerpts	10
	**Data analysis** - Process by which inferences, themes, etc., were identified and developed, including the researchers involved in data analysis; usually references a specific paradigm or approach; rationale**	10
	**Techniques to enhance trustworthiness** - Techniques to enhance trustworthiness and credibility of data analysis (e.g., member checking, audit trail, triangulation); rationale**	10-11

Results/findings

	**Synthesis and interpretation** - Main findings (e.g., interpretations, inferences, and themes); might include development of a theory or model, or integration with prior research or theory	15-17
	**Links to empirical data** - Evidence (e.g., quotes, field notes, text excerpts, photographs) to substantiate analytic findings	S9-13 Appendix

Discussion

	**Integration with prior work, implications, transferability, and contribution(s) to the field** - Short summary of main findings; explanation of how findings and conclusions connect to, support, elaborate on, or challenge conclusions of earlier scholarship; discussion of scope of application/generalizability; identification of unique contribution(s) to scholarship in a discipline or field	23-28
	**Limitations** - Trustworthiness and limitations of findings	27

Other

	**Conflicts of interest** - Potential sources of influence or perceived influence on study conduct and conclusions; how these were managed	29
	**Funding** - Sources of funding and other support; role of funders in data collection, interpretation, and reporting	29

	*The authors created the SRQR by searching the literature to identify guidelines, reporting standards, and critical appraisal criteria for qualitative research; reviewing

	the reference lists of retrieved sources; and contacting experts to gain feedback. The SRQR aims to improve the transparency of all aspects of qualitative research by providing clear standards for reporting qualitative research.<br><br>**The rationale should briefly discuss the justification for choosing that theory, approach, method, or technique rather than other options available, the assumptions and limitations implicit in those choices, and how those choices influence study conclusions and transferability. As appropriate, the rationale for several items might be discussed together.

S2 Appendix. PRISMA checklist

Section/topic	#	Checklist item	Reported on page #
TITLE			
Title	1	Identify the report as a systematic review, meta-analysis, or both.	1
ABSTRACT			
Structured summary	2	Provide a structured summary including, as applicable: background; objectives; data sources; study eligibility criteria, participants, and interventions; study appraisal and synthesis methods; results; limitations; conclusions and implications of key findings; systematic review registration number.	2-3
INTRODUCTION			
Rationale	3	Describe the rationale for the review in the context of what is already known.	5
Objectives	4	Provide an explicit statement of questions being addressed with reference to participants, interventions, comparisons, outcomes, and study design (PICOS).	6
METHODS			
Protocol and registration	5	Indicate if a review protocol exists, if and where it can be accessed (e.g., Web address), and, if available, provide registration information including registration number.	NA
Eligibility criteria	6	Specify study characteristics (e.g., PICOS, length of follow-up) and report characteristics (e.g., years considered, language, publication status) used as criteria for eligibility, giving rationale.	11-12
Information sources	7	Describe all information sources (e.g., databases with dates of coverage, contact with study authors to identify additional studies) in the search and date last searched.	11-12
Search	8	Present full electronic search strategy for at least one database, including any limits used, such that it could be repeated.	S5 Appendix
Study selection	9	State the process for selecting studies (i.e., screening, eligibility, included in systematic review, and, if applicable, included in the meta-analysis).	11-13
Data collection process	10	Describe method of data extraction from reports (e.g., piloted forms, independently, in duplicate) and any processes for obtaining and confirming data from investigators.	12-13

Data items	11	List and define all variables for which data were sought (e.g., PICOS, funding sources) and any assumptions and simplifications made.	12-13
Risk of bias in individual studies	12	Describe methods used for assessing risk of bias of individual studies (including specification of whether this was done at the study or outcome level), and how this information is to be used in any data synthesis.	NA
Summary measures	13	State the principal summary measures (e.g., risk ratio, difference in means).	NA
Synthesis of results	14	Describe the methods of handling data and combining results of studies, if done, including measures of consistency (e.g., I^2) for each meta-analysis.	NA

Section/topic	#	Checklist item	Reported on page #
Risk of bias across studies	15	Specify any assessment of risk of bias that may affect the cumulative evidence (e.g., publication bias, selective reporting within studies).	NA
Additional analyses	16	Describe methods of additional analyses (e.g., sensitivity or subgroup analyses, meta-regression), if done, indicating which were pre-specified.	NA
RESULTS			
Study selection	17	Give numbers of studies screened, assessed for eligibility, and included in the review, with reasons for exclusions at each stage, ideally with a flow diagram.	18; Fig.5
Study characteristics	18	For each study, present characteristics for which data were extracted (e.g., study size, PICOS, follow-up period) and provide the citations.	18-22
Risk of bias within studies	19	Present data on risk of bias of each study and, if available, any outcome level assessment (see item 12).	NA
Results of individual studies	20	For all outcomes considered (benefits or harms), present, for each study: (a) simple summary data for each intervention group (b) effect estimates and confidence intervals, ideally with a forest plot.	S17-23 Appendix
Synthesis of results	21	Present results of each meta-analysis done, including confidence intervals and measures of consistency.	NA
Risk of bias across studies	22	Present results of any assessment of risk of bias across studies (see Item 15).	NA
Additional analysis	23	Give results of additional analyses, if done (e.g., sensitivity or subgroup analyses, meta-regression [see Item 16]).	NA
DISCUSSION			

Summary of evidence	24	Summarize the main findings including the strength of evidence for each main outcome; consider their relevance to key groups (e.g., healthcare providers, users, and policy makers).	23-28
Limitations	25	Discuss limitations at study and outcome level (e.g., risk of bias), and at review-level (e.g., incomplete retrieval of identified research, reporting bias).	26
Conclusions	26	Provide a general interpretation of the results in the context of other evidence, and implications for future research.	28
FUNDING			
Funding	27	Describe sources of funding for the systematic review and other support (e.g., supply of data); role of funders for the systematic review.	29

S3 Appendix. Questionnaire used for the online survey

Information on the survey

The WHO **Collaborating Centre MNCH Trieste** is conducting, on request of **WHO EURO**, a survey on practices of antenatal screenings within the **53 countries** of the WHO European Region.

You have been identified as an expert in this field. We kindly ask you to answer the 20 questions of the survey. Time needed is less than **10-12 minutes**.

Please note that the questions aim at **identifying antenatal screening practices** in your country/institution (which may differ from what you do at your personal level).

WHY TO PARTECIPATE

Your help is very much appreciated; results will aid improving practices within the Region!

Survey findings will be published as a technical report and a scientific paper. We will acknowledge survey respondents in these publications.

DEADLINE: 18th February 2019

PRIVACY: Data are anonymized. Your identity will not be revealed.

1) Please select your country
    1) Albania
    2) Andorra
    3) Armenia
    4) Austria
    5) Azerbaijan
    6) Belarus
    7) Belgium
    8) Bosnia Herzegovina
    9) Bulgaria
    10) Croatia
    11) Cyprus
    12) Czech Republic
    13) Denmark
    14) Estonia
    15) Finland
    16) France
    17) Georgia

18) Germany
19) Greece
20) Hungary
21) Iceland
22) Ireland
23) Israel
24) Italy
25) Kazakhstan
26) Kyrgyzstan
27) Latvia
28) Lithuania
29) Luxembourg
30) Macedonia
31) Malta
32) Monaco
33) Montenegro
34) Netherlands
35) Norway
36) Poland
37) Portugal
38) Republic of Moldova
39) Romania
40) Russian Federation
41) San Marino
42) Serbia
43) Slovakia
44) Slovenia
45) Spain
46) Sweden
47) Switzerland
48) Tajikistan
49) Turkey
50) Turkmenistan
51) Ukraine
52) UK

53) Uzbekistan

2) Which of the following better describes the institution you are working in and your role? You can tick more than one field, if needed.

- Ministry of Health or other regulatory health authority (and working in a maternal health related field)
- Member of scientific society of obstetrician and gynecologist
- Research institute/University/EBM centre/UN agencies/other institution conducting research/implementation (working in maternal health)
- Doctor working in clinical care (of pregnant women)
- Other

3) Are there in your country official National guidelines on antenatal screenings?

- Yes, there are official national guidelines (recognized by MoH/local health authorities as "national")
- There are national guidelines, but they are not official
- No > go to question #7
- I am not sure/ I don't know

4) If there are national guidelines on antenatal screening, please add title/ or source/link / author

---

5) Are the official National guidelines updated based on recent evidence and covering all topics of antenatal screenings, as reflected in the tables in questions #11 and #12 (ie, covering the topic, independently from the recommendations)?

- Yes
- No
- I am not sure/ I don't know

6) Are the official National guidelines widely used?

- Yes
- No
- I am not sure/ I don't know

7) Are there other guidelines used? You can tick more than ones

- Guidelines developed by your own institution/s
- Guidelines develop by local (ie, based in your country) Scientific Societies/organization
- Guidelines developed by international Scientific Societies/organization (such as NICE, RCOG, or others)
- Guidelines developed by WHO
- All/most of the above

- None of the above

8) Overall, how many different guidelines (ie, from different organizations such as different Scientific Societies) do you believe are actually used in clinical practice within your country? **(please consider the national average, not only your personal practice)**

- From 1 to 3
- From 3 to 5
- More 5

9) Overall, when thinking at **different guidelines used in your county**, how would you rate the degree of heterogeneity in the recommendations among different guidelines? **(please consider the national average, not only your personal practice)**
   - Low (different guidelines having very similar recommendations)
   - Medium (different guidelines having some difference in recommendations)
   - High (different guidelines having high difference recommendations)

10) Overall, when thinking at **different institutions/hospitals in your country**, how would you rate the degree of heterogeneity in the above ANC screening practices among **different institutions/hospitals**?
   - Low (different institution having very similar practices)
   - Medium (different institution having some difference in practices)
   - High (different institution having high difference practices)

11) Are the following practices of antenatal screening used in your country/institution and how often?

Type of measurement/screening	Always/ nearly always	Sometimes	Rarely	Never	I am not sure, I believe there is high heterogeneity at country level
Weight measurement in all pregnant women at each visit					
Screening of anaemia in all pregnant women					
Screening of asymptomatic bacteriuria (ASB) in all pregnant women					
Screening for gestational diabetes in women with risk factors					
Screening of fetal growth by					

abdominal palpation with symphysis-fundal height (SFH) measurement					
HIV testing in all pregnant women					
Screening for syphilis in all pregnant women at an early stage					
Screening for tuberculosis (TB) in settings where there is high (prevalence in the general population is 100/100 000 population or higher)					
Ultrasound before 24 weeks for gestational age, multiple pregnancies and fetal anomalies					
Fetal echocardiography involving the four-chamber view and outflow tracts as part of fetal anomaly scan					
Serological screening for hepatitis B virus in all pregnant women					
Rubella susceptibility screening in all pregnant women early in antenatal care[1]					
Information of pregnant women younger than 25 years about the high prevalence of chlamydia infection in their age group, and provision of details of their local National Chlamydia Screening Programme.					
Testing for blood group and rhesus D status in early pregnancy					
Screening for atypical red-cell alloantibodies in early pregnancy and again at 28 weeks, in all					

[1] NICE withdrawn the recommendation on rubella after the online survey started, in late January 2019. Therefore, in the questionnaire the practice is still classified as "recommended", while in the results it is classified as "not recommended").

pregnant women					
Pre-conception counselling (supportive listening, advice-giving and information) and carrier testing in all women who are identified as being at higher risk of haemoglobinopathies					
Blood pressure measurement and urinalysis for protein at each antenatal visit to screen for pre-eclampsia					
Screening for Down's syndrome in all pregnant women					

12) Are the following practices of antenatal screening **used in your country** and how often?

Type of measurement/screening	Always/ nearly always	Sometimes	Rarely	Never	I am not sure, I believe there is high heterogeneity at country level
Daily fetal movement counting, such as with "count-to-ten" kick charts, as screening for fetal well-being (in all pregnant women)					
Antenatal cardiotocography as screening for fetal well-being (in all pregnant women)					
Ultrasound for monitoring fetal growth in the 3rd trimester (in all pregnant women)					
Doppler ultrasound examination of fetal blood vessels as screening for fetal well-being (in all pregnant women)					
Identification of asymptomatic bacterial vaginosis					
CMV screening (in all pregnant women)					
Hepatitis C screening (in all					

pregnant women)					
Toxoplasmosis screening (in all pregnant women)					
Group B streptococcus screening (in all pregnant women)					
Screening (cervical length) for preterm labour (without any symptom)					
Vaginal examination at each antenatal visits (without any symptom)					
Screening of thrombophilia in all pregnant women (without any risk factor)					
Screening of thyroid function (without any symptom)					
Amniocentesis or chorionic villus sampling for screening of chromosomic anomalies (fetal karyotype only) in all pregnant women					
Screening for pre-eclampsia in 1st trimester					
Noninvasive prenatal testing (NIPT) in all pregnant women					
Genetic fetal arrays					

13) Are there other antenatal screening used in your country?

- Yes
- No
- I don't know

14) If yes, what?

15) If there is high heterogeneity of practices within the country, what are the reasons behind? Please identify all of the following as "major" or "minor" reason. Thank you!

Type of measurement/screening	Major reason	Minor reason	Not at all a reason	I am not sure

Lack of national guidelines covering the item				
Lack of trust in the national guidelines				
Lack of adequate diffusion of the national guidelines				
Discrepancies between guidelines				
Discrepancies between guidelines and high-level experts' opinion				
Unclear or evolving evidence				
Lack of institutional protocols				
Lack of equipment or tools				
Lack of adequate knowledge or skills/training				
Lack of supervision				
Practice based on tradition				
Request from patients				
Fear of litigation				
Different practice in public health services vs private				
Commercial interest				

16) In your country was recently (last 5 years) conducted any national survey on ANC screening practices?

- Yes
- No
- I don't know

17) If yes, can you please provide the reference/s (author, year, other keywords for identification etc.) or the link to the report?

18) Is there any other screening on the horizon/being researched?

19) In your country in the last 15 years has any antenatal screening practice be suspended (ie, not more implemented) due to lack of evidence or funding or for other reasons?

- Yes
- No
- I don't know

20) If yes, on which specific antenatal screening/s and for what reasons/s?

S4 Appendix. Professional characteristics of KIs

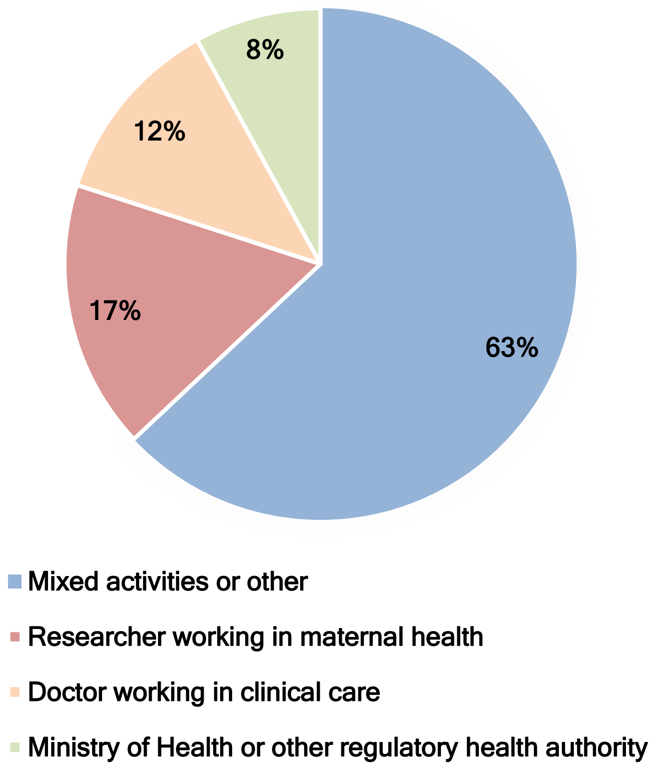

S5 Appendix. Search strategies for the literature review

PubMed	Date: 30 July 2019	Total retrieved:	1942
"Maternal Serum Screening Tests"[Mesh] OR ((maternal OR pregnancy OR antenatal OR prenatal) AND (screening OR testing OR diagnosis OR examination OR investigation OR test)) AND ("Italy"[Mesh] OR "Austria"[Mesh] OR "Russia"[Mesh] OR "Europe"[Mesh] OR "Europe, Eastern"[Mesh] OR "Czech Republic"[Mesh] OR "Slovakia"[Mesh] OR "Denmark"[Mesh] OR "France"[Mesh] OR "Georgia (Republic)"[Mesh] OR "Germany"[Mesh] OR "Greece"[Mesh] OR "Hungary"[Mesh] OR "Iceland"[Mesh] OR "Ireland"[Mesh] OR "Israel"[Mesh] OR "Luxembourg"[Mesh] OR "Malta"[Mesh] OR "Montenegro"[Mesh] OR "Netherlands"[Mesh] OR "Norway"[Mesh] OR "Poland"[Mesh] OR "Portugal"[Mesh] OR "Spain"[Mesh] OR "Turkey"[Mesh] OR "Ukraine"[Mesh] OR "England"[Mesh] OR Albania OR Andorra OR Armenia OR Azerbaijan OR Belarus OR Belgium OR "Bosnia Herzegovina" OR Bulgaria OR Croatia OR Cyprus OR Estonia OR Finland OR Kazakhstan OR Kyrgyzstan OR Latvia OR Lithuania OR Monaco OR "Republic of Moldova" OR Romania OR "San Marino" OR Serbia OR Sweden OR Switzerland OR Tajikistan OR "The former Yugoslav Republic of Macedonia" OR Turkmenistan OR Uzbekistan) Filters: Books and Documents; Consensus Development Conference; Editorial; Guideline; Legislation; Observational Study; Practice Guideline; Review; Systematic Reviews; published in the last 5 years; English			
Global health library	Date: 30 July 2019	Total retrieved:	2914
(tw:(antenatal screening)) OR ((tw:(maternal OR pregnancy OR prenatal)) AND (tw:(screening OR testing OR diagnosis OR examination OR investigation OR test))) AND (instance:"ghl") AND (mj:("Pregnancy" OR "Prenatal Diagnosis") AND clinical_aspect:("diagnosis") AND la:("en"))			
Web of Science (Core)	Date: 30 July 2019	Total retrieved:	3754
(TS=Antenatal screening* OR (TS=(maternal OR pregnancy OR prenatal) AND TS=(screening OR testing OR diagnosis OR examination OR investigation OR test)) AND (AD=(Italy OR Austria OR Russia OR Europe OR Czech Republic OR Slovakia OR Denmark OR France OR Georgia OR Germany OR Greece OR Hungary OR Iceland OR Ireland OR Israel OR Luxembourg OR Malta OR Montenegro OR Netherlands OR Norway OR Poland OR Portugal OR Spain OR Turkey OR Ukraine OR England OR Albania OR Andorra OR Armenia OR Azerbaijan OR Belarus OR Belgium OR Bosnia Herzegovina OR Bulgaria OR Croatia OR Cyprus OR Estonia OR Finland OR Kazakhstan OR Kyrgyzstan OR Latvia OR Lithuania OR Monaco OR Moldova OR Romania OR San Marino OR Serbia OR Sweden OR Switzerland OR Tajikistan OR Yugoslavia OR Macedonia OR Turkmenistan OR Uzbekistan) AND TS=(Italy OR Austria OR Russia OR Europe OR Czech Republic OR Slovakia OR Denmark OR France OR Georgia OR Germany OR Greece OR Hungary OR Iceland OR Ireland OR Israel OR Luxembourg OR Malta OR Montenegro OR Netherlands OR Norway OR Poland OR Portugal OR Spain OR Turkey OR Ukraine OR England OR Albania OR Andorra OR Armenia OR Azerbaijan OR Belarus OR Belgium OR Bosnia Herzegovina OR Bulgaria OR Croatia OR Cyprus OR Estonia OR Finland OR Kazakhstan OR Kyrgyzstan OR Latvia OR Lithuania OR Monaco OR Moldova OR Romania OR San Marino OR Serbia OR Sweden OR Switzerland OR Tajikistan OR Yugoslavia OR Macedonia OR Turkmenistan OR			

Uzbekistan))) AND LANGUAGE: (English) Indexes=SCI-EXPANDED, SSCI Timespan=Last 5 years			
Google	Date: 15 Jan 2019	Total retrieved:	2840

All these words: Screening Survey

Any of these words: maternal pregnancy antenatal prenatal None of these words: study genetic USA Australia Canada Zealand Caribbean China Africa

Language: English

Region: any region

Last update: 2015-2019

Terms appearing: In text of the page

All these words: Screening Guideline

Any of these words: maternal pregnancy antenatal prenatal

None of these words: study genetic USA Australia Canada Zealand Caribbean China Africa

Language: English

Region: any region

Last update: 2015-2019

Terms appearing: In text of the page

All these words: Screening Policy

Any of these words: maternal pregnancy antenatal prenatal

None of these words: study genetic USA Australia Canada Zealand Caribbean China Africa

Language: English

Region: any region

Last update: 2015-2019

Terms appearing: In text of the page

Limits: first 150 records per each search, exclusively English language

For each of the WHO EURO Region countries: Albania, Andorra, Armenia, Austria, Azerbaijan, Belarus, Belgium, Bosnia Herzegovina, Bulgaria, Croatia, Cyprus, Czech Republic, Denmark, Estonia, Finland, France, Georgia, Germany, Greece, Hungary, Iceland. Ireland, Israel, Italy, Kazakhstan, Kyrgyzstan, Latvia, Lithuania, Luxembourg, Macedonia, Malta, Monaco, Montenegro, Netherlands, Norway, Poland, Portugal, Republic of Moldova, Romania, Russian Federation, San Marino, Serbia, Slovakia, Slovenia, Spain, Sweden, Switzerland, Tajikistan, Turkey, Turkmenistan, Ukraine, United Kingdom, Uzbekistan

All these words: Screening Survey

Any of these words: maternal pregnancy antenatal prenatal None of these words: study genetic USA Australia Canada Zealand Caribbean China Africa

Last update: 2015-2019

Terms appearing: In text of the page

All these words: Screening Guideline
Any of these words: maternal pregnancy antenatal prenatal
None of these words: study genetic USA Australia Canada Zealand Caribbean China Africa
Region: Any region
Last update: 2015-2019
Terms appearing: In text of the page

All these words: Screening Policy
Any of these words: maternal pregnancy antenatal prenatal
None of these words: study genetic USA Australia Canada Zealand Caribbean China Africa
Region: any region
Last update: 2015-2019
Terms appearing: In text of the page

S6 Appendix. ANC practices compared between WHO and other reference guidelines

ANC practices (ordered as in original documents)	Recommended by WHO	Reference recommendations
Weight	Yes	Yes
Anaemia	Yes	Yes
Asymptomatic bacteriuria (ASB)	Yes	Yes
Gestational diabetes mellitus (GDM)	Yes	Yes
Human immunodeficiency virus (HIV)	Yes	Yes
Syphilis	Yes	Yes
Tuberculosis (TB)	Context-specific recommendation	Not included in the guidelines
Fetal Growth	Context-specific recommendation	Yes
Gestation age, multiple pregnancies and fetal anomalies ultrasound	Yes	Yes
Alloimmunization	Not included in the guidelines	Yes
Atypical red-cell alloantibodies	Not included in the guidelines	Yes
Haemoglobinopathies	Not included in the guidelines	Yes
Cardiac anomalies	Not included in the guidelines	Yes
Chromosomal abnormalities	Not included in the guidelines	Yes
Hepatitis B virus	Not included in the guidelines	Yes
Pre-eclampsia	Yes	Yes
**ANC practices (ordered as in the original document)**	**Not recommended by WHO**	**Not recommended by NICE/RCOG/ETA**
Daily fetal movement counting	Context-specific recommendation	Yes
Antenatal cardiotocography	Yes	Yes
Doppler ultrasound of fetal blood vessels	Yes	Yes
Vaginal examination	Not included in the	Yes

	guidelines	
Chromosomal abnormalities	Not included in the guidelines	Yes
Asymptomatic bacterial vaginosis	Not included in the guidelines	Yes
Chlamydia trachomatis	Not included in the guidelines	Yes
Cytomegalovirus	Not included in the guidelines	Yes
Hepatitis C virus	Not included in the guidelines	Yes
Rubella	Not included in the guidelines	Yes
Group B streptococcus	Not included in the guidelines	Yes
Toxoplasmosis	Not included in the guidelines	Yes
Pre-term birth	Not included in the guidelines	Yes
Fetal Growth	Not included in the guidelines	Yes
Thrombophilia	Not included in the guidelines	Yes (RCOG)
Thyroid function	Not included in the guidelines	Yes (ETA)

Abbreviations: ETA=European Thyroid Association, NICE=National Institute for Health and Care Excellence; RCOG=Royal College of Obstetricians and Gynecologists; WHO=World Health Organization

S7 Appendix. Details on the "recommended" ANC screenings by reference sources

ANC screening practices	Source	Recommendation (Number in original document)	Type of recommendation
Weight	WHO[2]	(A.1.1): Counselling about healthy eating and keeping physically active during pregnancy is recommended for pregnant women to stay healthy and to prevent excessive weight gain during pregnancy.	Recommended
	NICE[3]	(1.5.1.1): Maternal weight and height should be measured at the booking appointment, and the woman's body mass index should be calculated (weight [kg]/height[m]^2).<br>(1.5.1.2): Repeated weighing during pregnancy should be confined to circumstances in which clinical management is likely to be influenced.	Recommended
Anaemia	WHO[1]	(B.1.1): Full blood count testing is the recommended method for diagnosing anaemia in pregnancy. In settings where full blood count testing is not available, on-site haemoglobin testing with a haemoglobinometer is recommended over the use of the haemoglobin colour scale as the method for diagnosing anaemia in pregnancy.	Recommended
	NICE[2]	(1.6.1.1): Pregnant women should be offered screening for anaemia. Screening should take place early in pregnancy (at the booking appointment) and at 28 weeks when other blood screening tests are being performed. This allows enough time for treatment if anaemia is detected	Recommended
Asymptomatic bacteriuria (ASB)	WHO[1]	(B.1.2): Midstream urine culture is the recommended method for diagnosing asymptomatic bacteriuria (ASB) in pregnancy. In settings where urine culture is not available, on-site midstream urine Gram-staining is recommended over the use of dipstick tests as the	Recommended

[2] WHO recommendations on antenatal care for a positive pregnancy experience. World Health Organization, Geneva, 2016

[3] Antenatal care for uncomplicated pregnancies. CG62 Published date: March 2008 Last update February 2019

	NICE[2]	method for diagnosing ASB in pregnancy.<br>(1.8.1.1): Women should be offered routine screening for asymptomatic bacteriuria by midstream urine culture early in pregnancy. Identification and treatment of asymptomatic bacteriuria reduces the risk of pyelonephritis.	Recommended
Gestational diabetes mellitus (GDM)	WHO[1]	(B.1.4): Hyperglycaemia first detected at any time during pregnancy should be classified as either gestational diabetes mellitus (GDM) or diabetes mellitus in pregnancy, according to WHO criteria.<br>This is not a recommendation on routine screening for hyperglycaemia in pregnancy. It has been adapted and integrated from the WHO publication Diagnostic criteria and classification of hyperglycaemia first detected in pregnancy (2013)[5]	Recommended
	NICE[4]	1.2.2 Assess risk of gestational diabetes using risk factors in a healthy population. At the booking appointment, determine the following risk factors for gestational diabetes:<br>• BMI above 30 kg/m2<br>• previous macrosomic baby weighing 4.5 kg or above<br>• previous gestational diabetes<br>• family history of diabetes (first‑degree relative with diabetes)<br>• minority ethnic family origin with a high prevalence of diabetes.<br>Offer women with any one of these risk factors testing for gestational diabetes (see recommendations 1.2.5–1.2.7).	Recommended

[4] Diabetes in pregnancy: management from preconception to the postnatal period. NICE guideline 3. February 2015. nice.org.uk/guidance7ng3 Last updated: August 2015

5 World Health Organization. Diagnostic Criteria and Classification of Hyperglycaemia First Detected in Pregnancy. WHO/NMH/MND/13.2. World Health Organization, Geneva, 2013.

Human immunodeficiency virus (HIV)	WHO[1]	(B.1.7): In high-prevalence settings[6], provider-initiated testing and counselling (PITC) for HIV should be considered a routine component of the package of care for pregnant women in all antenatal care settings. In low-prevalence settings, PITC can be considered for pregnant women in antenatal care settings as a key component of the effort to eliminate mother-to-child transmission of HIV, and to integrate HIV testing with syphilis, viral or other key tests, as relevant to the setting, and to strengthen the underlying maternal and child health systems.[7]	Recommended
	NICE[2]	(1.8.7.1): Pregnant women should be offered screening for HIV infection early in antenatal care because appropriate antenatal interventions can reduce mother-to-child transmission of HIV infection.	Recommended
Syphilis	WHO[1]	(B.1.7): In high-prevalence settings[6], provider-initiated testing and counselling (PITC) for HIV should be considered a routine component of the package of care for pregnant women in all antenatal care settings. In low-prevalence settings, PITC can be considered for pregnant women in antenatal care settings as a key component of the effort to eliminate mother-to-child transmission of HIV, and to integrate HIV testing with syphilis, viral or other key tests, as relevant to the setting, and to strengthen the underlying maternal and child health systems.[8]	Recommended
	NICE[2]	(1.8.10.1): Screening for syphilis should be offered to all pregnant women at an early stage in antenatal care because treatment of syphilis is beneficial to the mother and baby.	Recommended
Tuberculosis (TB)	WHO[1]	(B.1.8): In settings where the tuberculosis (TB)	Context-specific

[6] High-prevalence settings are defined in the 2015 WHO publication Consolidated guidelines on HIV testing services as settings with greater than 5% HIV prevalence in the population being tested. Low-prevalence settings are those with less than 5% HIV prevalence in the population being tested. In settings with a generalized or concentrated HIV epidemic, retesting of HIV-negative women should be performed in the third trimester because of the high risk of acquiring HIV infection during pregnancy; please refer to Recommendation B.1.7 for details.

[7] Adapted and integrated from the *WHO publication Consolidated guidelines on HIV testing services (2015).*

[8] Adapted and integrated from the *WHO publication Consolidated guidelines on HIV testing services (2015).*

		prevalence in the general population is 100/100 000 population or higher, systematic screening for active TB should be considered for pregnant women as part of antenatal care.[9]	recommendation
Fetal Growth	WHO[1]	(B.2.2): Replacing abdominal palpation with symphysis-fundal height (SFH) measurement for the assessment of fetal growth is not recommended to improve perinatal outcomes. A change from what is usually practiced (abdominal palpation or SFH measurement) in a particular setting is not recommended.[10]	Context-specific recommendation
	NICE[2]	(1.10.1): Symphysis–fundal height should be measured and recorded at each antenatal appointment from 24 weeks.	Recommended
Gestation age, multiple pregnancies and fetal anomalies ultrasound	WHO[1]	(B.2.4): One ultrasound scan before 24 weeks of gestation (early ultrasound) is recommended for pregnant women to estimate gestational age, improve detection of fetal anomalies and multiple pregnancies, reduce induction of labour for post-term pregnancy, and improve a woman's pregnancy experience.[11]	Recommended
	NICE[2]	(1.2.6.1): Pregnant women should be offered an early ultrasound scan between 10 weeks 0 days and 13 weeks 6 days to determine gestational age and to detect multiple pregnancies. This will ensure consistency of gestational age assessment and reduce the incidence of induction of labour for prolonged pregnancy.<br>(1.7.1.1): Ultrasound screening for fetal anomalies should be routinely offered, normally between 18 weeks 0 days and 20 weeks 6 days.	Recommended

[9] Adapted and integrated from the WHO publication Systematic screening for active tuberculosis: principles and recommendations (2013).

[10] SFH measurement is routinely practiced in many ANC settings. Due to a lack of clear evidence of accuracy or superiority of either SFH measurement or clinical palpation to assess fetal growth, the GDG does not recommend a change of practice.

• The GDG agreed that there is a lack of evidence on SFH, rather than a lack of effectiveness, particularly in LMIC settings.

• Apart from false reassurance, which might occur with both SFH measurement and clinical palpation, there is no evidence of harm with SFH measurement.

• Research is needed to determine the role of SFH measurement in detecting abnormal fetal growth and other risk factors for perinatal morbidity (e.g. multiple pregnancy, polyhydramnios) in settings where antenatal ultrasound is not available.

11 Stakeholders should consider offering a late ultrasound scan to pregnant women who have not had an early ultrasound scan, for the purposes of identifying the number of fetuses, presentation and placental location.

Alloimmunization	NICE[2]	(1.6.2.1): Women should be offered testing for blood group and rhesus D status in early pregnancy.	Recommended
Atypical red-cell alloantibodies	NICE[2]	(1.6.2.3): Women should be screened for atypical red-cell alloantibodies in early pregnancy and again at 28 weeks, regardless of their rhesus D status.	Recommended
Haemoglobinopathies	NICE[2]	(1.6.3.3): Screening for sickle cell diseases and thalassaemias should be offered to all women as early as possible in pregnancy (ideally by 10 weeks). The type of screening depends upon the prevalence and can be carried out in either primary or secondary care.<br>(1.6.3.4): Where prevalence of sickle cell disease is high (fetal prevalence above 1.5 cases per 10,000 pregnancies), laboratory screening (preferably high‑performance liquid chromatography) should be offered to all pregnant women to identify carriers of sickle cell disease and/or thalassaemia.<br>(1.6.3.6): If the woman is identified as a carrier of a clinically significant haemoglobinopathy then the father of the baby should be offered counselling and appropriate screening without delay.	Recommended
Cardiac anomalies	NICE[2]	(1.7.1.5): Fetal echocardiography involving the four-chamber view of the fetal heart and outflow tracts is recommended as part of the routine anomaly scan[12]	Recommended
Chromosomal abnormalities	NICE[2]	(1.7.2.1): All pregnant women should be offered screening for Down's syndrome. Women should understand that it is their choice to embark on screening for Down's syndrome.	Recommended
Hepatitis B virus	NICE[2]	(1.8.5.1): Serological screening for hepatitis B virus should be offered to pregnant women so that effective postnatal interventions can be offered to infected women to decrease the risk of mother-to-child transmission.	Recommended
Pre-eclampsia	WHO[1]	Antenatal screening for pre-eclampsia is an essential part of good ANC. It is routinely	Recommended

[12] 1.7.1.6 Routine screening for cardiac anomalies using nuchal translucency is not recommended. [2008]

		performed by measuring maternal blood pressure and checking for proteinuria at each ANC contact[13]	
	NICE[2]	(1.9.2.1): Blood pressure measurement and urinalysis for protein should be carried out at each antenatal visit to screen for pre-eclampsia.	Recommended

Abbreviations: BMI=Body max index

[13] The GDG did not evaluate evidence or make a recommendation on this procedure, therefore, which it considers to be an essential component of Good Clinical Practice in ANC.

S8 Appendix. Details on the “not recommended” ANC screenings, by reference sources

ANC practices	Source	Recommendation (Number in original document)	Type of recommendation
Daily fetal movement counting	WHO[1]	(B.2.1): Daily fetal movement counting, such as with “count-to-ten” kick charts, is only recommended in the context of rigorous research.	Context-specific recommendation
	NICE[2]	(1.10.6): Routine formal fetal-movement counting should not be offered.	Not Recommended
Antenatal cardiotocography	WHO[1]	(B.2.3): Routine antenatal cardiotocography is not recommended for pregnant women to improve maternal and perinatal outcomes.	Not recommended
	NICE[2]	(1.10.8): The evidence does not support the routine use of antenatal electronic fetal heart rate monitoring (cardiotocography) for fetal assessment in women with an uncomplicated pregnancy and therefore it should not be offered.	Not recommended
Doppler ultrasound of fetal blood vessels	WHO[1]	(B.2.5): Routine Doppler ultrasound examination is not recommended for pregnant women to improve maternal and perinatal outcomes.	Not recommended
	NICE[2]	(1.10.3): Routine Doppler ultrasound should not be used in low-risk pregnancies	Not recommended
Vaginal examination	NICE[2]	(1.5.3.1): Routine antenatal pelvic examination does not accurately assess gestational age, nor does it accurately predict preterm birth or cephalopelvic disproportion. It is not recommended.	Not recommended
Chromosomal abnormalities by morphological scan	NICE[2]	(1.7.2.7) The routine anomaly scan (at 18 weeks 0 days to 20 weeks 6 days) should not be routinely used for Down's syndrome screening using soft markers. [2008]	Not recommended
Asymptomatic bacterial vaginosis	NICE[2]	(1.8.2.1): Pregnant women should not be offered routine screening for bacterial vaginosis because the evidence suggests that the identification and treatment of asymptomatic bacterial vaginosis does not lower the risk of preterm birth and other adverse reproductive outcomes.	Not recommended

Chlamydia trachomatis	NICE2	(1.8.3.2): Chlamydia screening should not be offered as part of routine antenatal care	Not recommended
Cytomegalovirus	NICE[2]	(1.8.4.1): The available evidence does not support routine cytomegalovirus screening in pregnant women and it should not be offered.	Not recommended
Hepatitis C virus	NICE[2]	(1.8.6.1): Pregnant women should not be offered routine screening for hepatitis C virus because there is insufficient evidence to support its clinical and cost effectiveness.	Not recommended
Rubella	NICE[2]	(1.8.8.1): Recommendation 1.8.8.1 on rubella screening has been withdrawn, as this is no longer offered on the NHS. See update information for more details[14]	Not recommended
Group B streptococcus	NICE[2]	(1.8.9.1): Pregnant women should not be offered routine antenatal screening for group B streptococcus because evidence of its clinical and cost effectiveness remains uncertain.	Not recommended
Toxoplasmosis	NICE[2]	(1.8.11.1): Routine antenatal serological screening for toxoplasmosis should not be offered because the risks of screening may outweigh the potential benefits.	Not recommended
Pre-term birth	NICE[2]	(1.9.3.1): Routine screening for preterm labour should not be offered.	Not recommended
Fetal Growth	NICE[2]	(1.10.2): Ultrasound estimation of fetal size for suspected large-for-gestational-age unborn babies should not be undertaken in a low-risk population.<br>(1.10.9): The evidence does not support the routine use of ultrasound scanning *(note of authors: for fetal growth)* after 24 weeks of gestation and therefore it should not be offered.	Not recommended
Thrombophilia	RCOG[15]	Clinicians should be aware that, at present, there is no evidence to support the use of pretest probability assessment in the management of acute VTE in pregnancy. Before anticoagulant therapy is commenced, blood should be taken for a full blood count, coagulation screen, urea and electrolytes, and liver function tests.	Not recommended

[14] https://www.nice.org.uk/guidance/cg62/chapter/update-information#update-information

[15] Thromboembolic Disease in Pregnancy and the Puerperium: Acute Management. Green-top Guideline No. 37b April 2015. Royal College of Obstetricians & Gynaecologist

		Performing a thrombophilia screen prior to therapy is not recommended.	
Thyroid function	European Thyroid Association [16]	Despite the beneficial effects of levothyroxine treatment on obstetric outcome and the fact that the previously recommended targeted approach to screening thyroid function will miss a large percentage of women with thyroid dysfunction, we do not recommend universal screening for SCH because of the lack of grade 1 evidence.	Not recommended

[16] Lazarus J, Brown RS, Daumerie C et al. 2014 European Thyroid Association Guidelines for the Management of Subclinical Hypothyroidism in Pregnancy and in Children. Eur Thyroid J 2014;3:76–94 doi: 10.1159/000362597

S9 Appendix. Findings of the online survey related to existence, type, and use of guidelines

Panel A: Are there in your country official National guidelines on antenatal screenings?

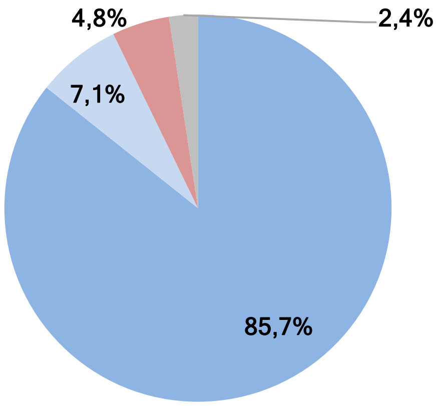

Yes: Armenia, Azerbaijan, Belarus, Belgium, Denmark, Estonia, Finland, France, Georgia, Germany, Hungary, Iceland, Ireland, Israel, Italy, Kazakhstan, Kyrgyzstan, Latvia, Lithuania, Luxembourg, Macedonia, Netherlands, Norway, Portugal, Republic of Moldova, Romania, Russia, San Marino, Slovenia, Spain, Sweden, Switzerland, Turkey, Ukraine, United Kingdom, Uzbekistan

Yes, but not officially recognised: Albania, Czech Repub

No: Croatia, Malta

Unknown: Greece

Panel B: Are the official National guidelines updated based on recent evidence and covering all topics of antenatal screenings?

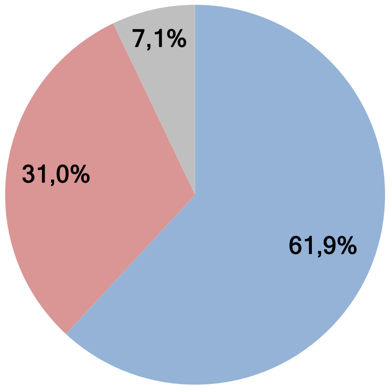

Yes: Armenia, Belarus, Belgium, Czech Republic, Denmark, Estonia, Finland, Georgia, Germany, Hungary, Ireland, Israel, Kazakhstan, Lithuania, Luxembourg, Netherlands, Norway, Poland, Portugal, Republic of Moldova, Romania, Spain, Sweden, Turkey, United Kingdom, Uzbekistan

No: Albania, Azerbaijan, Croatia, France, Iceland, Italy, Kyrgyzstan, Latvia, Macedonia, Malta, Russia, Slovenia, Ukraine

Unknown: Greece, Switzerland, San Marino

Panel C: Are the official National guidelines widely used?

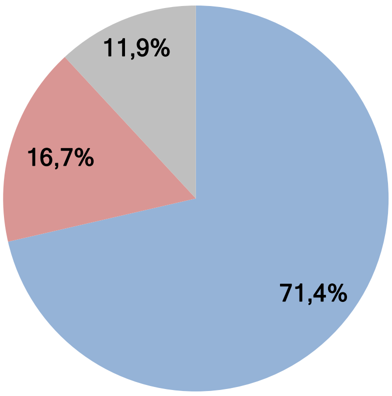

Yes: Armenia, Azerbaijan, Belarus, Belgium, Czech Republic, Denmark, Estonia, Finland, France, Georgia, Germany, Hungary, Iceland, Ireland, Israel, Italy, Latvia, Lithuania, Luxembourg, Macedonia, Netherlands,

Panel D: Are there other guidelines used?

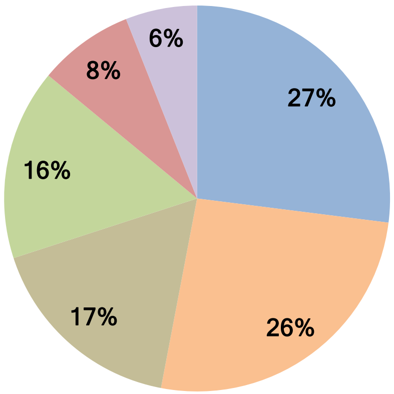

Guidelines developed by international Scientific Societies/organization

Guidelines develop by local Scientific Societies/organization

Norway, Poland, Republic of Moldova, Russia, Slovenia, Spain, Sweden, Ukraine, United Kingdom

No: Croatia, Kazakhstan, Kyrgyzstan, Malta, Romania, Turkey and Uzbekistan

Unknown: Albania, Greece, Portugal, Switzerland, San Marino

Guidelines developed by WHO

Guidelines developed by own institution/s

None of the above

All/most of the above

Panel E: Overall, when thinking at different guidelines used in your country, how would you rate the degree of heterogeneity in the recommendations among different guidelines?

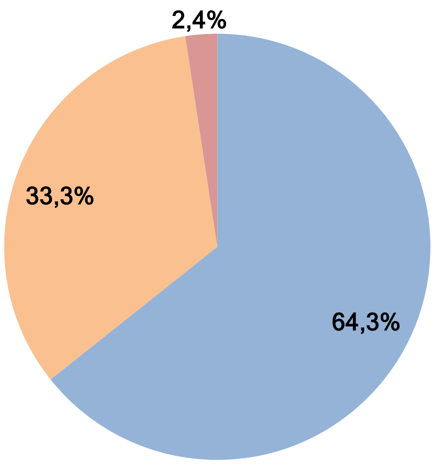

Low: Armenia, Azerbaijan, Belarus, Czech Republic, Denmark, Finland, France, Georgia, Germany, Hungary, Iceland, Ireland, Israel, Kyrgyzstan, Latvia, Lithuania, Luxembourg, Malta, Netherlands, Norway, Portugal, Republic of Moldova, Romania, Russia, San Marino, Sweden, Ukraine

Medium: Albania, Belgium, Croatia, Estonia, Greece, Italy, Macedonia, Poland, Slovenia, Spain, Switzerland, Turkey, United Kingdom, Uzbekistan

High: Kazakhstan

Panel F: In your country in the last 15 years has any antenatal screening practice be suspended?

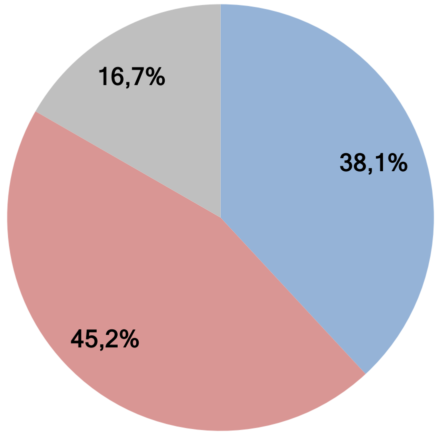

Yes: Albania, Belarus, Belgium, Croatia, Denmark, Estonia, Georgia, Hungary, Italy, Latvia, Netherlands, Republic of Moldova, Russia, Sweden, Switzerland, Uzbekistan

No: Azerbaijan, France, Germany, Greece, Iceland, Ireland, Israel, Lithuania, Luxembourg, Macedonia, Malta, Norway, Poland, Portugal, Slovenia, Spain, Turkey, Ukraine, United Kingdom

Unknown: Armenia, Czech Republic, Finland, Kazakhstan, Kyrgyzstan, Romania, San Marino

S10 Appendix. Other examples of ANC screening utilised at country level, reported by KIs

Mood disorders	Norway, Slovenia, Sweden
First trimester ultrasound screening (12w)	Belgium, Estonia
Cervical neoplasia	Belarus, Portugal
Nuchal translucency test gestational age (GA) 1-12+, anomaly scan GA 19-21	Denmark, Estonia
Gonorrhoea test	Belarus
Non-invasive prenatal test	Belgium
Screening for Vitamin D	Georgia
Biometry	Sweden
Hypothyroidism	Uzbekistan

S11 Appendix. ANC screening practices “on the horizon”, as reported by KIs

Innovative screening of pre-eclampsia	Denmark, Estonia, Switzerland, Uzbekistan
Non-invasive prenatal test	Estonia, Russia, Spain, Hungary
Research ongoing regarding screening on gestational diabetes	Denmark, Slovenia
Rhesus D fetal blood type	Belgium
Maternal cardiovascular profiling in the first trimester of pregnancies	Belgium
Intrauterine growth restriction (IUGR) screening (different criteria)	Estonia
Cancer Screening (not further clarified)	Georgia
Molecular fetal karyotyping (array)	Italy
Thyroid function in pregnancy	Macedonia
Innovative screening methods for cystic fibrosis	Uzbekistan
Thrombophilia	Uzbekistan

S12 Appendix. Factors affecting heterogeneity of ANC screenings, as reported by KIs

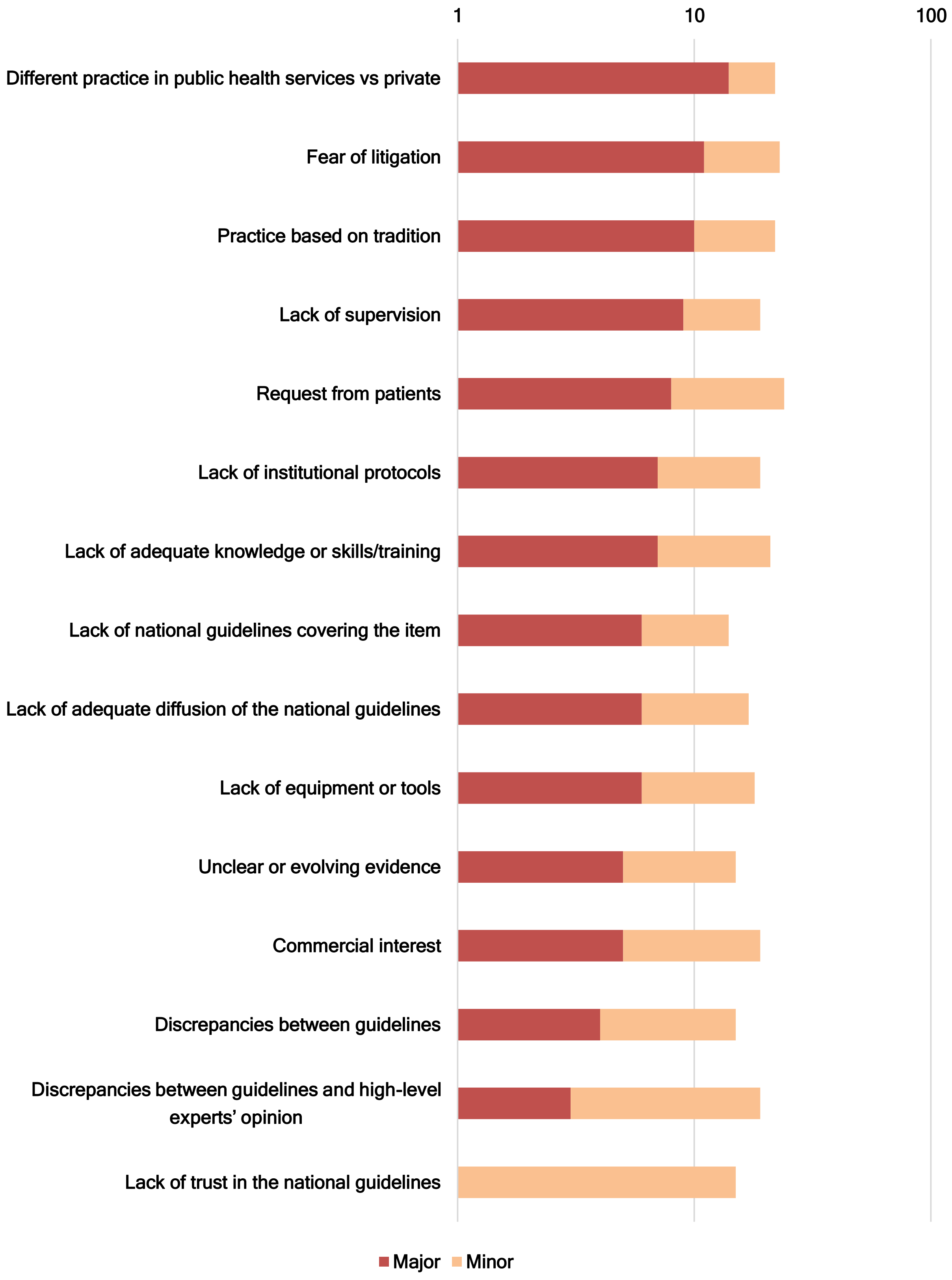

S13 Appendix. ANC screenings suspended in the last 15 years, as reported by KIs

CMV	Belgium, Switzerland
NIPT for special listed cases	Croatia, Estonia
Digital vaginal examination	Belgium
Toxoplasmosis screening every trimester	Belgium
Tripletest	Denmark
TORCH	Georgia
Bacterial vaginosis	Georgia
Maternal serum alfa-fetoprotein (AFP) measurement	Hungary
Ultrasound in the third trimester	Italy
Urine proteins at each visit	Italy
Rubella screening among vaccinated women	Netherlands
Routine vaginal smear	Republic of Moldova
Routine pelviometry	Republic of Moldova
2nd trimester biochemical screening	Russia
1st trimester screening for chromosomal anomalies	Sweden
Combined Ultrasound and Biochemical screening for chromosomal anomalies	Sweden
Fetal heart 5 planes	Sweden
Thrombophilia	Uzbekistan

Abbreviations: CMV=cytomegalovirus; NIPT=noninvasive prenatal testing; TORCH=Toxoplasma gondii, other viruses (HIV, measles, and so on), rubella (German measles), cytomegalovirus, and herpes simplex.

S14 Appendix. References included in the systematic review

CROSS-SECTIONAL STUDIES

1. Ministry of health: division of sexual, reproductive, children and youth health. Report on Prenatal diagnosis. Pre-Natal Diagnostic practices performed in health services in 2011.
2. Petersson K, Lindkvist M, Persson M, Conner P, ÅhmanA, Mogren I. Prenatal diagnosis in Sweden 2011 to 2013—a register-based study. BMC Pregnancy and Childbirth (2016) 16:365
3. Dansk Føtalmedicinsk Database. National årsrapport 2016
4. Deans ZC, Allen S, Jenkins L, Khawaja F, Hastings RJ, Mann K, Patton SJ, Sistermans EA, Chitty LS. Recommended practice for laboratory reporting of non-invasive prenatal testing of trisomies 13, 18 and 21: a consensus opinion. Prenatal Diagnosis 2017, 37, 699–704
5. Lewis C, Hill M, Chitty LS. Offering non-invasive prenatal testing as part of routine clinical service. Can high levels of informed choice be maintained? Prenatal Diagnosis 2017, 37:1130–1137.
6. Benhalima K, Van Crombrugge P, Devlieger R, Verhaeghe J, Verhaegen A, De Catte L, Mathieu C. Screening for pregestational and gestational diabetes in pregnancy: a survey of obstetrical centers in the northern part of Belgium. Diabetology & Metabolic Syndrome 2013, 5:66
7. Benhalima K, Mathieu C, Van Assche A, Dammc P, Devlieger R, Mahmoodd T, Dunne F. Survey by the European Board and College of Obstetrics and Gynaecology on screening for gestational diabetes in Europe. European Journal of Obstetrics & Gynecology and Reproductive Biology 201 (2016) 197–202
8. Pintaudi B, Fresa R, Dalfrà M, Marcone T, Dodesini A, Napoli A, Bonomo M. Level of implementation of guidelines on screening and diagnosis of gestational diabetes: A national survey. Diabetes research and clinical practice 113(2016)48-52
9. Bell R, Hayes L, Pasecinic N, Heslehurst N. Implementation of national screening guidelines for gestational diabetes: A national survey of maternity units in England. Diabetes Research and Clinical Practice 146(2018)58-66
10. Technical report: Antenatal screening for HIV, hepatitis B, syphilis and rubella susceptibility in the EU/EEA. European Centre for Disease Prevention and Control, 2016
11. Aebi-Popp K, Kahlert C, Rauch A, Mosimann B, Baud D, Low N, Surbek D. Heterogeneity in testing practices for infections during pregnancy: national survey across Switzerland. Swiss Med Wkly. 2016;146:w14325
12. Balla E, Donder G. Features of syphilis seropositive pregnant women raising alarms in Hungary, 2013–2016. European Journal of Obstetrics & Gynecology and Reproductive Biology 228 (2018) 274–278
13. Surveillance de la santé périnatale au Luxembourg 2011-2012-2013. Ministère de la santé 2016.
14. Enquête nationale périnatale Rapport 2016
15. Surveillance de la santé périnatale au Luxembourg 2014-2015-2016. Ministère de la santé

16. Halle K, Fjose M, Kristjansdottir H, Bjornsdottir A, Getz L, Tomasdottir M and Sigurdsson J. Use of pregnancy ultrasound before the 19th week scan: an analytical study based on the Icelandic Childbirth and Health Cohort. BMC Pregnancy and Childbirth (2018) 18:512
17. Kullinger M, Granfors M, Kieler H, Skalkidou A. Adherence to Swedish national pregnancy dating guidelines and management of discrepancies between pregnancy dating methods: a survey study. Reproductive Health (2019) 16:95 https://doi.org/10.1186/s12978-019-0760-3

GUIDELINES

18. Societé suisse de gynécologie et obstétrique. Abandonment of toxoplasmosis screening during pregnancy - brief summary of justification. Lettre d'experts No 31 (2010)
19. Societé suisse de gynécologie et obstétrique. Prevention of neonatal group B early-stage streptococcal infection. Avis d'Experts No 19 (2012)
20. Nordic Federation of Societies of Obstetrics and Gynecology. Group B streptococcus in pregnancy and delivery. 2014
21. Societé suisse de gynécologie et obstétrique. Cytomegalovirus (CMV) and pregnancy. Avis d'Experts No 47 (2016)
22. Hughes RG, Brocklehurst P, Steer PJ, Heath P, Stenson BM on behalf of the Royal College of Obstetricians and Gynaecologists. Prevention of early-onset neonatal group B streptococcal disease. Green-top Guideline No. 36. BJOG 2017;124:e280–e305
23. Biskupska M, Kujawa A, Wysocki J. Preventing congenital toxoplasmosis - implementation of clinical practice guidelines. Ginekologia Polska 2018, vol. 89, no. 7, 388–392
24. Societé suisse de gynécologie et obstétrique. Pregnancy and HIV: prevention of vertical transmission of HIV. Avis d'Experts No 60 (remplace No 20) (2018)
25. NHS Plymouth Hospitals 2018. Group B Streptococcus. CLI.MAT.GUI.689.7
26. NHS Royal Cornwall Hospitals 2018. Human Immunodeficiency Virus (HIV) Maternity Clinical guideline
27. Seedat F, Geppert J, Stinton C, Patterson J, Freeman K, Johnson S, Fraser H, Stewart Brown C, Uthman O, Tan B, Robinson E, McCarthy ND, Clarke A, Marshall J, Visintin C, Mackie A, Taylor-Phillips S. Universal antenatal screening for group B streptococcus may cause more harm than good. BMJ 2019;364:l463
28. Bevan D, White A, Marshall J, Catherine Peckham C. Modelling the effect of the introduction of antenatal screening for group B Streptococcus (GBS) carriage in the UK. BMJ Open 2019;9:e024324. doi:10.1136/bmjopen-2018-024324
29. Paris L, Imbert S. Serological monitoring and prevention of toxoplasmosis during pregnancy. Rev Prat. 2019 Mar;69(3):291-296
30. Wolf B. Tuberculosis in Pregnancy-A summary. Geburtsh Frauenheilk 2019; 79: 358–364
31. Nordic Federation of Societies of Obstetrics and Gynecology. Prenatal diagnostic. 2014
32. Ministry of Health of Macedonia. Screening for fetal chromosomic abnormalities. 2015

33. Ustav E, Asser K, Haldre K, Muru K, Ridnõi K, Buts K, Rajasalu L, Reimand T. Sünnieelse diagnostika juhend: loote kromosoomhaiguste sõeluurimine ja diagnoosimine. Loote ultraheliuuringud. Versioon 4. Kinnitatud 04.03.2016
34. Lou S, Petersen OB, Jørgensen FS, Lund I, Kjærgaard S, Danish Cytogenetic Central Registry Study Group, Vogel I. National screening guidelines and developments in prenatal diagnoses and live births of Down syndrome in 1973–2016 in Denmark. Acta Obstetricia et Gynecologica Scandinavica 97 (2018) 195–203
35. NHS 2018. NHS public health functions agreement 2018-19. NHS Fetal Anomaly Screening Programme - Screening for Down's Syndrome, Edwards' Syndrome and Patau's Syndrome (Trisomy 21, 18 & 13). NHS England Gateway Number: 07837
36. NHS Mid Essex Hospital Services 2018. Amniocentesis for antenatal diagnosis. Clinical guidelines register n° 08045
37. Smith R, Evans A. Guideline for Antenatal Screening for Trisomy 21, Trisomy 18 and Trisomy 13. NHS Norfolk and Norwich University Hospital 2018. Trust Docs ID: 836
38. Payne L, Ulyett H, Matthews L. Down's, Patau's and Edwards' Syndrome (Trisomies) Screening Guideline. NHS University Hospitals of Leicester 2018. Guideline Register No: C6/2001
39. State of Israel Ministry of Health. Screening Tests for the Detection of Down Syndrome. Available: https://www.health.gov.il/English/Topics/Genetics/checks/during_pregnancy/Pages/screening_tests.aspx
40. State of Israel Ministry of Health. Placenta Chorion Test. Available: https://www.health.gov.il/English/Topics/Genetics/checks/during_pregnancy/Pages/placenta.aspx
41. State of Israel Ministry of Health. Amniotic fluid test. Available: https://www.health.gov.il/English/Topics/Genetics/checks/during_pregnancy/Pages/Amniotic_fluid.aspx
42. Portuguese Society of Endocrinology, Diabetes and Metabolism; Portuguese Society of Diabetology; Portuguese Society of Obstetrics and Maternal-Fetal Medicine; Section of Neonatology of the Portuguese Society of Pediatrics. Relatório de Consenso sobre a Diabetes e Gravidez. January 2011
43. Societé suisse de gynécologie et obstétrique. Screening of Gestational Diabetes. Avis d'Experts No 37 (2011)
44. Benhalima K, Devlieger R, Van Assche A. Screening and management of gestational diabetes. Best Practice & Research Clinical Obstetrics and Gynaecology 29 (2015) 339e349
45. Benhalima K, Damm P, Van Assche A, Mathieu C, Devlieger R, Mahmood T, Dunne F. Screening for gestational diabetes in Europe: where do we stand and how to move forward? A scientific paper commissioned by the European Board & College of Obstetrics and Gynaecology (EBCOG). European Journal of Obstetrics & Gynecology and Reproductive Biology 201 (2016) 192–196

46. Ellenberg A, Sarvilinna N, Gissler M, Ulander V. New guidelines for screening, diagnosing, and treating gestational diabetes – evaluation of maternal and neonatal outcomes in Finland from 2006 to 2012. Acta Obstetricia et Gynecologica Scandinavica 96 (2017) 372–381
47. Wielgos M, Bomba-Opon D, Czajkowski K, Wender-Ozegowska E, Hod M. Towards a European Consensus on Gestational Diabetes Mellitus: A Pragmatic Guide for Diagnosis, Management, and Care. The Polish Diabetes in Pregnancy Study Group and FIGO. Ginekologia Polska 2017, vol. 88, no. 1, 46–49
48. Schäfer-Graf UM, Gembruch U, Kainer F, Groten T, Hummel S, Hösli I, Grieshop M, Kaltheuner M, Bührer C, Kautzky-Willer A, Laubner K, Bancher-Todesca D. Gestational Diabetes Mellitus (GDM) – Diagnosis, Treatment and Follow-Up. Guideline of the DDG and DGGG. Geburtsh Frauenheilk 2018; 78: 1219–1231
49. SFOG Guideline 2014 Obstetric Ultrasound
50. Ministry of Health of Macedonia. Ultrasound survey on pregnancy. 2015
51. Society and College of Radiographers and British Medical Ultrasound Society (BMUS). Guidelines for Professional Ultrasound Practice. 2015
52. Ministry of Health of Luxembourg. Ultrasound and monitoring. 2016. Available: http://sante.public.lu/fr/prevention/grossesse-maternite/suivi-prenatal/echo-ctg/index.html
53. German Society for Gynecology and Obstetrics 2013. Diagnostik und Therapie hypertensiver Schwangerschaftserkrankungen. AWMF-Register Nr. 015/018 Klasse: S1
54. Nordic Federation of Societies of Obstetrics and Gynecology. Hypertensive disorders of pregnancy and eclampsia. 2014
55. Institute of Obstetricians and Gynaecologists, Royal College of Physicians of Ireland, Clinical Strategy and Programmes Division, Health Service Executive. The diagnosis and management of severe pre-eclampsia and eclampsia. 2016
56. Societé suisse de gynécologie et obstétrique. Specification of the risks of preeclampsia in the 1st trimester. Avis d'Experts No 57 (10.02.2019)
57. White J, Qureshi H, Massey E, Needs M, Byrne G, Daniels G, Allard S. Guideline for blood grouping and red cell antibody testing in pregnancy. Trasfusion Medicine 2016
58. Dutch Ministry of Health. Draaiboek Prenatale Screening Infectieziekten en Erytrocytenimmunisatie versie 6.0, April 2018
59. Societé suisse de gynécologie et obstétrique. Diagnosis and treatment of iron deficiency anemia during pregnancy and postpartum. Avis d'Experts No 22 (2009)
60. Api O, Breyman C, Cetiner M, Demir C, Ecder T. Diagnosis and treatment of iron deficiency anemia during pregnancy and the postpartum period: Iron deficiency anemia working group consensus report. Turk J Obstet Gynecol 2015;12:173-81

61. Danish Society of Obstetrics and Gynecology 2016. Anaemia and iron deficiency in pregnancy and postpartum
62. NHS Basildon and Thurrock University Hospitals 2019. Iron deficiency anaemia in pregnancy. Document reference/register n° 08011
63. Danish Society of Obstetrics and Gynecology 2014. Small for Gestational age (SGA), Fetal Growth restriction (FGR), Intrauterine Growth Retardation (IUGR)
64. Vayssiere C, Sentilhes L, Ego A, Bernard C. Fetal growth restriction and intra-uterine growth restriction: guidelines for clinical practice from the French College of Gynaecologists and Obstetricians. European Journal of Obstetrics & Gynecology and Reproductive Biology 193 (2015) 10–18
65. Norwegian Society of Obstetrics and Gynecology. Intrauterine growth restriction. June 2016
66. Institute of Obstetricians and Gynaecologists, Directorate of Quality and Strategy Health Service Executive. Fetal growth restriction – recognition, diagnosis & management. 2017
67. NFOG 2014. Door-step cardiotocography (CTG)
68. Institute of Obstetricians and Gynaecologists, Royal College of Physicians of Ireland, HSE Clinical Care Programme in Obstetrics and Gynaecology and Irish Haematology Society Venous thromboprophylaxis in pregnancy. 2016
69. SFOG Guideline from Endokrin-ARG. Thyroid Disease in the Perinatal Period. 2014
70. Italian Ministry of Health. Gravidanza fisologica. 2008
71. Ministry of Health of Lithuania. Metodika antenataline prieziura. 2014
72. Russian Ministry of Health. Order of the Ministry of Health of Russia dated November 12, 2012. № 572 n
73. Spanish Ministry of Health. Guía de práctica clínica de atención en el embarazo y puerperio. 2014
74. Belgian Health Care Knowledge Centre. What are the recommended clinical assessment and screening tests during pregnancy? http://kce.fgov.be/content/about-copyrights-for-kce-reports. 2015
75. Danish Ministry of Health. Anbefalinger for svangreomsorgen 2013
76. Ministry of Health of Georgia. Antenatal screening for a physiological pregnancy 2017
77. Ministry of Health of Iceland. Meðgönguvernd heilbrigðra kvenna í eðlilegri meðgöngu 2008
78. Ministry of Health Care Ukraine. The organization of ambulatory obstetric and gynecological care in Ukraine. July 15, 2011 Decree N417
79. Ministry of Health of Latvia. Dzemdību palīdzības nodrošināšanas kārtība. 2006
80. Norwegian Ministry of Health. National Guideline for antenatal care (short version). 2005
81. German Ministry of Health 2016. Richtlinien des Gemeinsamen Bundesausschusses über die ärztliche Betreuung während der Schwangerschaft und nach der Entbindung. "Mutterschafts-Richtlinien"
82. Haute Autorité de santé. Suivi et orientation des femmes enceintes en fonction des situations à risque identifiées 2016
83. Turkish Ministry of Health. Prenatal Care Management Guide. 2014

84. Dutch Ministry of Health. Checklist on prenatal care. 2018
85. Hungarian Ministry of Helath 2014. Decree on Pregnant Care.
86. Nordic Federation of Societies of Obstetrics and Gynecology. Antenatal care. 2014
87. Ministry of Health of Republic of Azerbaijan. FİZİOLOJİ HAMİLƏ LİYİ OLAN QADINLARA ANTENATAL QULLUQ ÜZRƏ KLİNİK PROTOKOL. 2013
88. Ministry of Health of Moldova. Perinatology National Guideline. 2006
89. Ministry of Health of Slovenia. Preventive health care at the primary level (unofficial consolidated text No. 13). 2018
90. Ministry of Health of Luxembourg. Laboratory analyzes. 2015. Available: http://sante.public.lu/fr/prevention/grossesse-maternite/suivi-prenatal/analyses/index.html
91. State of Israel Ministry of Health. Monitoring of Pregnancy and Medical Examinations During Pregnancy. Available: https://www.health.gov.il/English/Topics/Pregnancy/during/examination/Pages/permanent.aspx
92. Ministry of Health of Luxembourg. Gynecological consultations and examinations. 2015. Available: http://sante.public.lu/fr/prevention/grossesse-maternite/suivi-prenatal/consultation-gynecologique/index.html
93. NHS 2017. Screening tests for you and your baby. www.gov.uk/topic/population-screening-programmes
94. Republic of Albania, Ministry of Health. Strategic Document on Reproductive Health 2009-2015
95. Italian Ministry of Health. Screening prenatale non invasivo basato sul DNA (Non Invasive Prenatal Testing – NIPT). 2015
96. SFOG Guidelines 2016. Analysis of foetal DNA in the woman's blood: non-invasive prenatal testing (NIPT) for trisomy 13, 18 and 21
97. Societé suisse de gynécologie et obstétrique. Prenatal genetic screening: new model. (Supplements of the FOPH information sheet of 7.7.2015). Avis d'Experts No 45
98. Sieroszewski P, Wielgos M, Radowicki S. Cell-free fetal DNA testing in prenatal diagnosis: Recommendations of the Polish Gynecological Society and the Polish Human Genetics Society. European Journal of Obstetrics & Gynecology and Reproductive Biology 214 (2017) 190–191
99. Societé suisse de gynécologie et obstétrique. Noninvasive prenatal assessment of the risk of fetal aneuploidy. Avis d'Experts No 52
100. Kozlowski P, Burkhardt T, Gembruch U, Gonser M, Kähler C, Kagan KO, von Kaisenberg C, Klaritsch P, Merz E, Steiner H, Tercanli S, Vetter K, Schramm T. DEGUM, ÖGUM, SGUM and FMF Germany Recommendations for the Implementation of First-Trimester Screening, Detailed Ultrasound, Cell-Free DNA Screening and Diagnostic Procedures. Ultraschall in Med 2019; 40: 176–193

101. Institute of Obstetricians and Gynaecologists, Royal College of Physicians of Ireland, Clinical Strategy and Programmes Division, Health Service Executive. Parvovirus B19 Exposure/Infection during pregnancy. 2017
102. Institute of Obstetricians and Gynaecologists, Royal College of Physicians of Ireland, and the National Clinical Programme in Obstetrics and Gynaecology. Bacterial infections specific to pregnancy. 2018
103. Institute of Obstetricians and Gynaecologists, Royal College of Physicians of Ireland, Clinical Strategy and Programmes Division, Health Service Executive. Chickenpox in pregnancy. 2018
104. Institute of Obstetricians and Gynaecologists, Royal College of Physicians of Ireland, Clinical Strategy and Programmes Division, Health Service Executive. Listeriosis in pregnancy. 2018
105. German Association for the Control of Viral Diseases, Society for Virology, German Society for Gynecology and Obstetrics, Professional Association of Gynecologists, German Society for Pediatric Infectiology. Labordiagnostik schwangerschaftsrelevanter Virusinfektionen. AWMF Registernummer 0093/001
106. ISUOG. Practice Guidelines: role of ultrasound in screening for and follow-up of pre-eclampsia. Ultrasound Obstet Gynecol 2018
107. Orosz L, Orosz G, Veress L, Dosa D, Arany I, Fabiand A, Medved L, Pap K, Karanyi Z, Toth, Poka R, Thang NG, Torok O. Screening for preeclampsia in the first trimester of pregnancy in routine clinical practice in Hungary, Journal of Biotechnology Volume 300, 20 July 2019, Pages 11-19 https://doi.org/10.1016/j.jbiotec.2019.04.017

POLICIES

108. Special Report: Prenatal Screening Policies in Europe. Eurocat 2005
109. Special Report: Prenatal Screening Policies in Europe. Eurocat. 2010
110. Scottish Government. A Refreshed Framework for Maternity Care in Scotland. APS Group Scotland DPPAS11078 (01/11)
111. Ministry of Health of Portugal. Diagnosis and management of Gestational Diabetes. Standard directorate-general for health, number 007/2011

S15 Appendix. Records included in the systematic review, by country

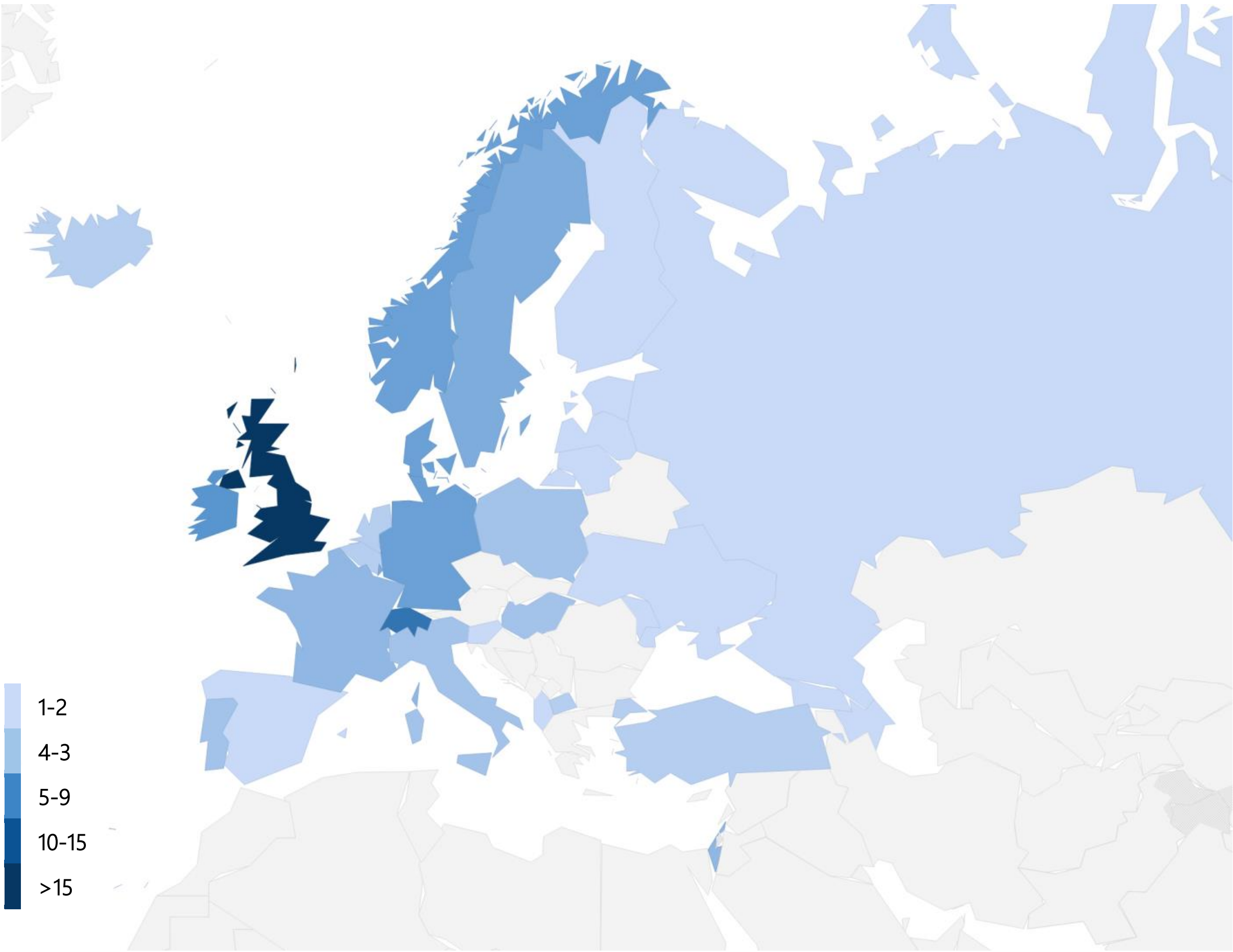

S16 Appendix. Characteristics of guidelines on ANC screenings identified by the systematic review (N=90)

References Guidelines and Policies	N (%)
Topic specific	52 (57.8)
Multi-topics	25 (27.8)
Screenings "on the horizon"	13 (14.4)
**Written by**	
MoH	37 (41.1)
Scientific societies	33 (36.7)
Research groups without direct endorsement of any organization/institution	12 (13.3)
Research Institutes	8 (8.9)
**Year published/released**	
During or after 2015	62 (68.8)
Before 2015	28 (31.1)
**By place of origin**	
United Kingdom	13 (14.4)
Switzerland	9 (10)
Ireland	7 (7.8)
Germany; Norway	6 (6.6)
Denmark	5 (5.6)
Israel	4 (4.4)
France; Luxembourg; Poland; Sweden; EU countries	3 (3.3)
Hungary; Italy; Macedonia; Netherlands; Turkey	2 (2.2)
Albania; Azerbaijan; Belgium; Estonia; Finland; Georgia; Iceland; Latvia; Lithuania; Moldova; Portugal; Russia; Slovenia; Spain; Ukraine	1 (1.1)

Abbreviations: MoH=Ministry of Health; EU=European Union

S17 Appendix. Characteristics of multi-topic guidelines identified by the systematic review and comparison with reference recommendations

ANC practices covered N (%)	Country	Author/ Year	Guidelines	ANC practices in concordance with reference guidelines* N (%)
30 (93.7)	Italy	MoH 2011	Physiological pregnancy	24 (75)
29 (90.6)	Lithuania	MoH 2014	Methodology for antenatal care	26 (81.2)
29 (90.6)	Russia	MoH 2012	Order of the Ministry of Health of Russia № 572 n	13 (40.6)
28 (87.5)	Spain	MoH 2014	Clinical practice guide attention in pregnancy and puerperium	24 (75)
28 (87.5)	Belgium	KCE 2015	What are the recommended clinical assessment and screening tests during pregnancy?	22 (68.7)
27 (84.3)	Denmark	MoH 2013	Recommendations for pregnant women	24 (75)
27 (84.3)	Georgia	MoH 2017	Antenatal screening for a physiological pregnancy	22 (68.7)
26 (81.2)	Iceland	MoH 2008	Pregnancy protection for healthy women in normal pregnancy	24 (75)
26 (81.2)	Ukraine	MoH 2011	The organization of ambulatory obstetric and gynecological care in Ukraine	24 (75)
23 (71.8)	Latvia	MoH 2006	Procedures for Provision of Maternity Assistance	20 (62.5)
23 (71.8)	Norway	MoH 2005	National Guideline for antenatal care (short version)	18 (56.2)
21 (65.6)	Germany	MoH 2016	Guidelines of the Joint Federal Committee about the medical care during pregnancy and after delivery ("Maternity guidelines")	17 (53.1)
19 (59.3)	France	MoH 2016	Monitoring and orientation of pregnant women based on identified risk situations	18 (56.2)
17 (53.1)	Turkey	MoH 2014	Prenatal Care Management Guide (in Turkish)	14 (43.7)
15 (46.8)	Netherlands	MoH 2018	Checklist on prenatal care	15 (46.8)
15 (46.8)	Hungary	MoH 2014	Decree on Pregnant Care	13 (40.6)

15 (46.8)	Norway	NFOG 2014	Antenatal care	13 (40.6)
14 (43.7)	Azerbaijan	MoH 2013	Women with physiological pregnancy. Antenatal care. Clinical protocol	13 (40.6)
13 (40.6)	Moldova	MoH 2006	Perinatology National Guideline	12 (37.5)
13 (40.6)	Slovenia	MoH 2018	Preventive health care at the primary level (unofficial consolidated text No. 13)	12 (37.5)
13 (40.6)	Luxembourg	MoH 2015	Laboratory analyzes	11 (34.3)
12 (37.5)	Israel	MoH 2019	Monitoring of Pregnancy and Medical Examinations During Pregnancy	11 (34.3)
8 (25)	Luxembourg	MoH 2015	Gynecological consultations and examinations	7 (21.8)
7 (21.8)	UK	NHS 2017	Screening tests for you and your baby	7 (21.8)
5 (15.6)	Albania	MoH 2015	Strategic document on reproductive health	5 (18.7)

*Numerator=ANC practices covered and in concordance with reference guidelines. Denominator=all 32 recommendations of the reference guidelines

Abbreviations: MoH=Ministry of Health; NHS=National Health Service; KCE=Belgian Health Care Knowledge Centre

S18 Appendix. Correlation among concordance rate and year of publication of multi-topic guidelines identified by the systematic review

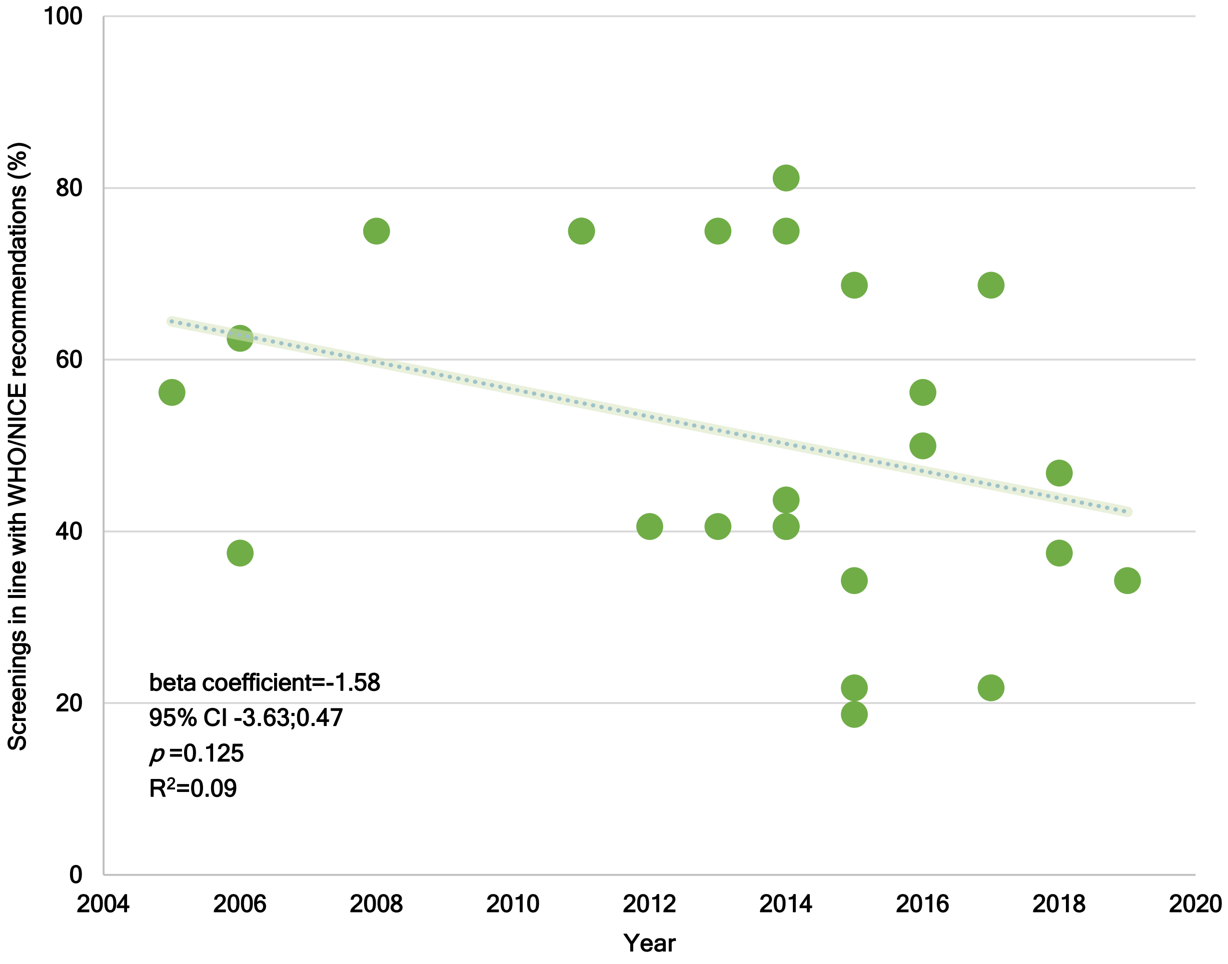

S19 Appendix. Characteristics of topic-specific guidelines identified by the systematic review and comparison with reference recommendations

ANC practices covered	Author year	Country	ANC practice covered N (%)	ANC practices in concordance with reference guidelines N (%)
Infectious diseases (Group B streptococcus, Toxoplasmosis, HIV, CMV, Tubercolosis)	Societé suisse de gynécologie et obstétrique 2010	Switzerland	13 (25)	10 (76.9)
	Societé suisse de gynécologie et obstétrique 2012	Switzerland		
	NFOG 2014	Norway		
	Societé suisse de gynécologie et obstétrique 2016	Switzerland		
	Royal College of Obstetricians & Gynaecologists 2017	UK		
	Biskupska 2018	Poland		
	Societé suisse de gynécologie et obstétrique 2018	Switzerland		
	NHS Plymouth Hospitals 2018	UK		
	NHS Royal Cornwall Hospitals 2018	UK		
	Seedat 2019	UK		
	Bevan 2019	UK		
	Paris 2019	France		
	Wolf 2019	Germany		
Chromosomal abnormalities	NFOG 2014	Norway	11 (21.2)	11 (100)
	MoH 2015	Macedonia		
	Ustav 2016	Estonia		
	Lou 2018	Denmark		
	NHS 2018	UK		
	NHS Mid Essex Hospital Services 2018	UK		
	NHS Norfolk and Norwich University Hospital 2018	UK		
	NHS University Hospitals of Leicester 2018	UK		
	MoH 2019	Israel		
	MoH 2019	Israel		
	MoH 2019	Israel		

Gestational Diabetes	SPEDM; SPD; SPOMMF; Section of Neonatology of the Portuguese Society of Pediatrics 2011	Portugal	7 (13.5)	6 (85.7)
	Societé suisse de gynécologie et obstétrique 2011	Switzerland		
	Benhalima 2015	Europe		
	EBCOG 2016	Europe		
	Ellenberg 2016	Finland		
	Polish Gynecological Society 2017	Poland		
	German Society of Gynecology and Obstetrics; Germany Diabetes Society 2018	Germany		
Ultrasound	SFOG 2014	Sweden	4 (7.7)	3 (75)
	MoH 2015	Macedonia		
	Society and College of Radiographers and BMUS 2015	UK		
	MoH 2016	Luxembourg		
Pre-eclampsia	German Society for Gynecology and Obstetrics 2013	Germany	4 (7.7)	4 (100)
	NFOG 2014	Norway		
	Institute of Obstetricians and Gynaecologists 2016	Ireland		
	Societé suisse de gynécologie et obstétrique 2019	Switzerland		
Aneamia	Societé suisse de gynécologie et obstétrique 2009	Switzerland	4 (7.7)	4 (100)
	Api 2015	Turkey		
	Danish Society of Obstetrics and Gynecology 2016	Denmark		
	NHS Basildon and Thurrock University Hospitals 2019	UK		
Fetal Growth	Danish Society of Obstetrics and Gynecology 2014	Denmark	4 (7.7)	3 (75)
	French College of Gynaecologists and Obstetricians 2015	France		
	Norwegian Society of Obstetrics and Gynecology 2016	Norway		
	Institute of Obstetricians and Gynaecologists 2017	Ireland		
Alloimmunization	British Blood Transfusion Society 2016	UK	2 (3.8)	2 (100)

	MoH 2018	Netherlands		
**CTG**	NFOG 2014	Denmark	1 (1.9)	1 (100)
**Thrombophilia**	Institute of Obstetricians and Gynaecologists 2016	Ireland	1 (1.9)	1 (100)
**Thyroid**	SFOG 2014	Sweden	1 (1.9)	0 (0)
		**TOT**	**52**	**45 (86.5)**

Abbreviations: CMV=cytomegalovirus; CTG=Cardiotocography; EBCOG=European Board & College of obstetrics and gynaecology; HIV=Human immunodeficiency virus; NFOG=Nordic Federation of Societies of Obstetrics and Gynecology; NICE= National Institute for Clinical Excellence; NHS=National Health Service; SFOG=Swedish Society of Obstetrics and Gynecology; SPD=Portuguese Society of Diabetology; SPEDM= Portuguese Society of Endocrinology, Diabetes and Metabolism; SPOMMF=Portuguese Society of Obstetrics and Maternal-Fetal Medicine

S20 Appendix. ANC practices identified as “on the horizon” by the systematic review

ANC screenings	Author year	Country	Topic covered	N
Non-invasive prenatal test (NIPT)	MoH 2015	Italy	NIPT	6
	SFOG 2016	Sweden	NIPT	
	Societé suisse de gynécologie et obstétrique 2016	Switzerland	NIPT	
	Polish Gynecological Society 2017	Poland	NIPT	
	Societé suisse de gynécologie et obstétrique 2018	Switzerland	NIPT	
	Kozlowski 2019	Germany	NIPT	
Alternative screenings for pre-eclampsia	ISUOG 2018	Europe	Ultrasound use for pre-eclampsia screening	2
	Orosz 2019	Hungary	Pre-eclampsia risk calculation during first-trimester	
Infectious diseases	Institute of Obstetricians and Gynaecologists 2017	Ireland	Parvovirus B19	5
	Institute of Obstetricians and Gynaecologists 2018	Ireland	Bacterial infections specific to pregnancy	
	Institute of Obstetricians and Gynaecologists 2018	Ireland	Chickenpox	
	Institute of Obstetricians and Gynaecologists 2018	Ireland	Listeriosis	
	DVV, GfV, DGGG, Professional Association of Gynecologists, DPGI 2014	Germany	Viral infections	

Abbreviations: DGGG=German Society for Gynecology and Obstetrics; DPGI=German Society for Pediatric Infectiology; DVV= German Association for the Control of Viral Diseases; GfV=German Society for Virology; MoH=Ministry of Health; SFOG=Swedish Society of Obstetrics and Gynecology; ISUOG=International Society of Ultrasound in Obstetrics and Gynecology

S21 Appendix. Number of cross-sectional studies on ANC screening practices identified by the systematic review (N=17)

References survey	N (%)
**Author**	
MoH	4 (23.5)
Scientific societies/European center	2 (11.8)
Research groups without direct endorsement of any organization/institution	11 (64.7)
**Year published**	
By 2015	15 (88.2)
Before 2015	2 (11.8)
**By place of origin**	
EU countries	3 (17.6)
Luxembourg; Sweden; United Kingdom	2 (11.8)
Belgium; Denmark; France; Hungary; Iceland; Italy; Portugal; Switzerland	1 (5.9)

Abbreviations: MoH=Ministry of Health; EU=European Union

S22 Appendix. Characteristics of cross-sectional studies on ANC screening practices identified by the systematic review

Recommended practices	Year/Author	Country	Title	Sample	Reported coverage of ANC practices[1]
Chromosomal abnormalities	2013 MoH	Portugal	Pre-Natal Diagnostic Activities performed in health services in 2011	37 hospitals in Portugal	97.3%
	2016 Petersson	Sweden	Prenatal diagnosis in Sweden 2011 to 2013 - a register-based study	284,789 pregnancies from Swedish Pregnancy Register	33%
	2016 MOH	Denmark	The Danish Fetal Medical Database 2016	17 departments in Denmark	90.6%
	2017 MoH	France	National Perinatal Survey Report 2016	13,894 women pregnant women attended ANC services in France	88.2%
Diabetes	2013 Benhalima	Belgium	Screening for pregestational and gestational diabetes in pregnancy: a survey of obstetrical centers in the northern part of Belgium	65 obstetric centers in Belgium	67%
	2016 Benhalima	Europe	Survey by the European Board and College of Obstetrics and Gynaecology on screening for gestational diabetes in Europe	28 EU countries	64.3%

	2016 Pintaudi	Italy	Level of implementation of guidelines on screening and diagnosis of gestational diabetes: A national survey	122 diabetic centers in Italy	82%
	2017 MoH	France	National Perinatal Survey Report 2016	13,894 women pregnant women attended ANC services in France	73.2%
	2018 Bell	UK	Implementation of national screening guidelines for gestational diabetes: A national survey of maternity units in England	113 of NHS units in England	81%
HIV	2016 European Centre for Disease Prevention and Control	Europe	Antenatal screening for HIV, hepatitis B, syphilis and rubella susceptibility in the EU/EEA	26 EU countries	92.3%
	2016 Aebi-Popp	Switzerland	Heterogeneity in testing practices for infections during pregnancy: national survey across Switzerland	537 clinicians in Switzerland	94.7%
HBV	2016 European Centre for Disease Prevention and Control	Europe	Antenatal screening for HIV, hepatitis B, syphilis and rubella susceptibility in the EU/EEA	26 EU countries	88.5%

	Year/Author	Country	Title	Sample	Reported coverage of ANC practices[1]
	2016 Aebi-Popp	Switzerland	Heterogeneity in testing practices for infections during pregnancy: national survey across Switzerland	537 clinicians in Switzerland	96.5%
Syphilis	2016 European Centre for Disease Prevention and Control	Europe	Antenatal screening for HIV, hepatitis B, syphilis and rubella susceptibility in the EU/EEA	26 EU countries	100%
	2016 Aebi-Popp	Switzerland	Heterogeneity in testing practices for infections during pregnancy: national survey across Switzerland	537 clinicians in Switzerland	80.4%
Ultrasound	2018 Halle	Iceland	Use of pregnancy ultrasound before the 19th week scan: an analytical study based on the Icelandic Childbirth and Health Cohort	1111 women attending prenatal care at primary care in Iceland	95%
	2019 Kullinger	Sweden	Adherence to Swedish national pregnancy dating guidelines and management of discrepancies between pregnancy dating methods: a survey study.	38 units in Sweden	50%
Not recommended practices	Year/Author	Country	Title	Sample	Reported coverage of ANC practices[1]

Rubella	2016 European Centre for Disease Prevention and Control	Europe	Antenatal screening for HIV, hepatitis B, syphilis and rubella susceptibility in the EU/EEA	26 EU countries	53.8%
GBS	2016 Aebi-Popp	Switzerland	Heterogeneity in testing practices for infections during pregnancy: national survey across Switzerland	537 clinicians in Switzerland	98%
Toxoplasmosis	2016 Aebi-Popp	Switzerland	Heterogeneity in testing practices for infections during pregnancy: national survey across Switzerland	537 clinicians in Switzerland	24.1%
Data not available	Year/Author	Country	Title	Sample	Reported coverage of ANC practices[1]
Surveillance of perinatal health	2016 MOH	Luxembourg	Surveillance of perinatal health in Luxembourg 2011-2012-2013	19498 pregnant women attended ANC services in Luxembourg	No data available
	2019 MOH	Luxembourg	Surveillance of perinatal health in Luxembourg 2014-2015-2016	20315 pregnant women attended ANC services in Luxembourg	No data available
Chromosomal abnormalities	2017 Deans	Europe	Laboratory reporting of non-invasive prenatal testing of trisomies 13, 18 and 21: a consensus opinion	121 registered laboratories	No data available
	2017 Lewis	UK	Offering non-invasive prenatal testing as part of routine clinical	6 antenatal clinics in UK	No data available

			service. Can high levels of informed choice be maintained?		
Syphilis	2018 Balla	Hungary	Features of syphilis seropositive pregnant women raising alarms in Hungary, 2013–2016	49,965 pre-screened pregnant women in Hungary	No data available

[1] ANC screening practices "recommended" and "not recommended" by reference guidelines

S23 Appendix. WHO Research Priorities on ANC

A. Nutritional interventions

1. What are the effects, feasibility, acceptability and equity implications of healthy eating and exercise interventions in LMICs?
2. Can an intervention package with standardized guidance on nutrition be developed that is evidence-based, sustainable, reproducible, accessible and adaptable to different cultural settings?
3. Research is needed at country level to better understand the context-specific etiology of under-nutrition. Do alternatives to energy and protein supplements, such as cash or vouchers for pregnant women, or improved local and national food production and distribution, lead to improved maternal and perinatal outcomes?
4. What is the most effective, acceptable and feasible regimen of recommended supplements (iron, calcium and folic acid)? Could micronutrients be combined into a single, or slow-release, formulation? To what extent do iron and calcium (or zinc) supplements compete for absorption?
5. What is the most cost-effective iron compound and formulation (coated versus not) in terms of benefits and side effects?
6. Can a rapid, portable, less invasive, and field-friendly test for iron deficiency anaemia be developed?
7. Are there haemoconcentration risks associated with haemoglobin concentrations of more than 130 g/L in pregnancy?
8. What are the biological mechanisms underlying the relationships among calcium supplementation, preeclampsia, HELLP syndrome (haemolysis, elevated liver enzymes, low platelet count) and preterm birth?
9. What is the minimal dose and optimal commencement schedule for calcium supplementation to achieve a positive effect on pre-eclampsia and preterm birth?
10. What is the effect of zinc supplementation on maternal outcomes (e.g. infections) and perinatal outcomes (e.g. preterm birth, SGA, neonatal infections, perinatal morbidity)? What is the optimal dose of zinc supplementation in pregnancy, particularly in zinc-deficient populations with no food fortification strategy in place?
11. Does vitamin C reduce PROM and improve maternal and perinatal outcomes?
12. Does vitamin D increase the risk of preterm birth when it's combined with calcium?

B. Maternal and fetal assessment

13. Can better and more cost–effective on-site tests to diagnose anaemia be developed?
14. What are the effects of on-site urine testing (dipsticks or Gram stain) with antibiotic treatment for ASB versus urine testing plus culture confirmation of urine test, followed by ASB treatment if indicated, on pregnancy and other relevant outcomes, including equity, acceptability, feasibility and antimicrobial resistance?
15. Can better on-site tests to diagnose ASB be developed to improve accuracy and feasibility of ASB testing and reduce overtreatment of ASB? What is the threshold prevalence of ASB at which targeted testing and treatment rather than universal testing and treatment might be a more effective strategy?
16. Which strategies to enquire about and manage IPV are the most effective? Do interventions to enquire about IPV have an impact on ANC attendance? Can interventions focusing on partners prevent IPV? Does enquiry about IPV (with appropriate referral) have an impact on maternal and perinatal outcomes?

17. What is the prevalence of GDM and diabetes mellitus in pregnancy, according to the new criteria, in various populations and ethnic groups? What are the best screening strategies for GDM and what are the prevalence thresholds at which these are cost-effective?
18. What is the effect of daily fetal movement counting, such as the use of "count-to-ten" kick charts, in the third trimester of pregnancy on perinatal outcomes in LMICs?
19. What are the effects and accuracy of SFH measurement to detect abnormal fetal growth and other risk factors for perinatal morbidity (e.g. multiple pregnancy, polyhydramnios) in settings without routine ultrasound?
20. Can a single routine Doppler ultrasound examination of fetal blood vessels for all pregnant women in the third trimester accurately detect or predict pregnancy complications, particularly IUGR and pre-eclampsia, and lead to improved pregnancy outcomes?

C. Preventive measures

21. What are the effects of prophylactic antibiotics to prevent RUTI in pregnancy, compared to monitoring with use of antibiotics only when indicated, on maternal infections, perinatal morbidity and antimicrobial drug resistance?
22. What is the prevalence of Rh alloimmunization and associated poor outcomes among pregnant women in LMIC settings? Can cost-effective strategies be developed to manage this condition in LMICS and improve equity?

D. Interventions for common physiological symptoms

23. What is the prevalence of common physiological symptoms among pregnant women in low-resource settings, and can the offer of treatment of these symptoms reduce health inequality, improve ANC coverage and improve women's pregnancy experiences?
24. What is the etiology of leg cramps in pregnancy, and does treatment with magnesium and/or calcium relieve symptoms?

E. Health systems interventions to improve utilization and quality of ANC

25. What should be included in women-held case notes, and how can discrepancies across different records be reduced to improve quality of care?
26. What is the pathway of influence of midwife-led continuity of care (MLCC)? Is it specifically the continuity, the provider–client relationship or the midwifery philosophy that leads to better health outcomes and maternal satisfaction? Can this effect be replicated with other cadres of health-care providers, e.g. auxiliary nurse midwives, nurses, family doctors, etc.? How can ANC in LMICs be structured to incorporate the active ingredients of MLCC, particularly in settings where the number of midwives is very limited?
27. What are the effects, feasibility and resource implications of MLCC in LMICs? Which models are most feasible (i.e. caseload or team models)? Can a continuity model for group ANC be developed for settings where other MLCC models are not feasible?
28. Can a group ANC model be developed for LMICs, to provide guidance on the optimal group size, frequency and content of group ANC contacts?
29. Is group ANC acceptable (data should include the views of women who decline to participate), feasible and cost-effective in LMIC settings?

30. Are mixed models (group and individual ANC) feasible and acceptable, and are there benefits to mixed models?
31. What are the effects of group ANC on maternal and perinatal health outcomes, coverage outcomes (ANC contacts and facility-based births), and women's and providers' experiences?
32. Should women with complicated pregnancies also be offered group ANC, for the communication and social support aspects, in addition to receiving specialist care?
33. How acceptable and feasible are mixed-gender community mobilization groups? What are the optimal methods for community-based interventions to improve communication and support for pregnant women and adolescent girls; to improve integration of community-based mobilization efforts with health systems; and to ensure continuity of care with home visits? What are the mechanisms of effect of these interventions?
34. Can the 2016 WHO ANC model with a minimum of eight contacts impact the quality of ANC in LMICs, and what is the effect on health, values, acceptability, resources, feasibility and equity parameters?

ANC: antenatal care; ASB: asymptomatic bacteriuria; GDM: gestational diabetes mellitus; IPV: intimate partner violence; LMICs: low- and middle-income countries; MLCC: midwife-led continuity of care; PROM: prelabour rupture of membranes;
RUTI: recurrent urinary tract infections; SFH: symphysis-fundal height; SGA: small for gestational age
